# Drug-associated progressive multifocal leukoencephalopathy: a clinical, radiological, and cerebrospinal fluid analysis of 326 cases

**DOI:** 10.1007/s00415-016-8217-x

**Published:** 2016-07-11

**Authors:** Roderick P. P. W. M. Maas, Annemarie H. G. Muller-Hansma, Rianne A. J. Esselink, Jean-Luc Murk, Clemens Warnke, Joep Killestein, Mike P. Wattjes

**Affiliations:** 1 Department of Neurology, Donders Institute for Brain, Cognition, and Behaviour, Radboud University Medical Center, Route 935, PO Box 9101, 6500 HB Nijmegen, The Netherlands; 2 Netherlands Pharmacovigilance Center Lareb, ‘s-Hertogenbosch, The Netherlands; 3 Department of Medical Microbiology, University Medical Center Utrecht, Utrecht, The Netherlands; 4 Department of Neurology, Medical Faculty, Heinrich Heine University, Düsseldorf, Germany; 5 Department of Neurology, VU Medical Center, Amsterdam, The Netherlands; 6 Department of Radiology and Nuclear Medicine, VU Medical Center, Amsterdam, The Netherlands

**Keywords:** Progressive multifocal leukoencephalopathy, Medication, Drugs, Side effect, Adverse event

## Abstract

The implementation of a variety of immunosuppressive therapies has made drug-associated progressive multifocal leukoencephalopathy (PML) an increasingly prevalent clinical entity. The purpose of this study was to investigate its diagnostic characteristics and to determine whether differences herein exist between the multiple sclerosis (MS), neoplasm, post-transplantation, and autoimmune disease subgroups. Reports of possible, probable, and definite PML according to the current diagnostic criteria were obtained by a systematic search of PubMed and the Dutch pharmacovigilance database. Demographic, epidemiologic, clinical, radiological, cerebrospinal fluid (CSF), and histopathological features were extracted from each report and differences were compared between the disease categories. In the 326 identified reports, PML onset occurred on average 29.5 months after drug introduction, varying from 14.2 to 37.8 months in the neoplasm and MS subgroups, respectively. The most common overall symptoms were motor weakness (48.6 %), cognitive deficits (43.2 %), dysarthria (26.3 %), and ataxia (24.1 %). The former two also constituted the most prevalent manifestations in each subgroup. Lesions were more often localized supratentorially (87.7 %) than infratentorially (27.4 %), especially in the frontal (64.1 %) and parietal lobes (46.6 %), and revealed enhancement in 27.6 % of cases, particularly in the MS (42.9 %) subgroup. Positive JC virus results in the first CSF sample were obtained in 63.5 %, while conversion after one or more negative outcomes occurred in 13.7 % of cases. 52.2 % of patients died, ranging from 12.0 to 83.3 % in the MS and neoplasm subgroups, respectively. In conclusion, despite the heterogeneous nature of the underlying diseases, motor weakness and cognitive changes were the two most common manifestations of drug-associated PML in all subgroups. The frontal and parietal lobes invariably constituted the predilection sites of drug-related PML lesions.

**Electronic supplementary material:**

The online version of this article (doi:10.1007/s00415-016-8217-x) contains supplementary material, which is available to authorized users.

## Introduction

Progressive multifocal leukoencephalopathy (PML) is a JC virus (JCV) related demyelinating disorder of the central nervous system that occurs almost exclusively in immunocompromised patients [1]. It is characterized histopathologically by a lytic infection of oligodendrocytes, astrocytes, and/or neurons in the white matter, cortical gray matter, and/or gray-white matter junction giving rise to a plethora of clinical phenotypes [2]. Quintessential radiological lesions are hyperintense on T2-weighted and FLAIR images and hypointense on T1-weighted images. In 2013, diagnostic criteria have been established which include clinical, imaging, laboratory, and histopathological features. Prerequisite for a definite diagnosis of PML is either the presence of characteristic pathoanatomic findings in a biopsy specimen or a combination of the appropriate clinical symptoms, radiological features, and the detection of JCV DNA in cerebrospinal fluid (CSF) [3]. Cases that do not fulfill these criteria might be classified as possible or probable PML and may be missed in official statistics.

**Fig. 1:**
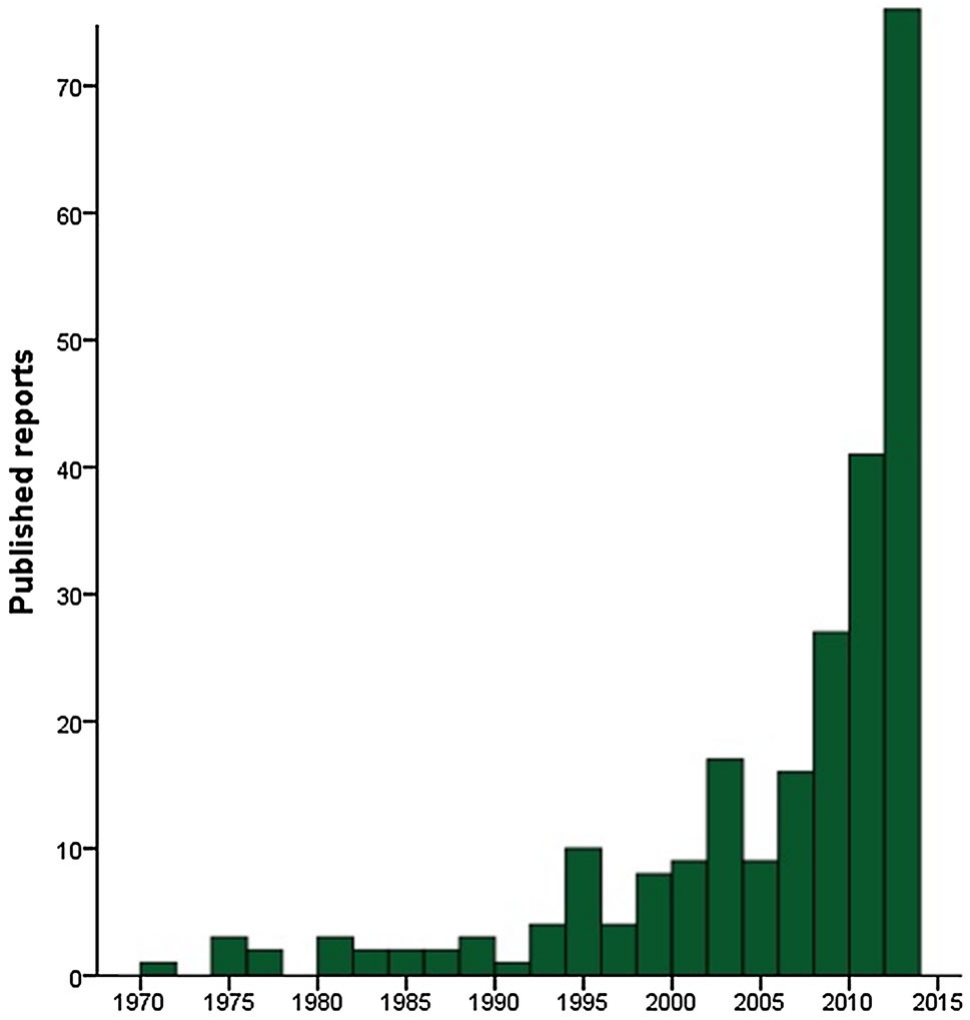
Number of published reports on drug-associated PML throughout the years, as included in this paper

PML has traditionally been associated with an intrinsically compromised immune system. In the past few years, a considerable number of immunosuppressive drugs has been approved by the authorities and implemented into clinical practice to broaden the therapeutic spectrum of a variety of medical conditions [4–6]. Consequently, medication-associated PML has become an increasingly prevalent clinical entity, as reflected by an exponential increase in the number of published cases (Fig. 1). Because of the frequently poor prognosis of PML, it is of paramount importance to establish this diagnosis at an early stage. However, a comprehensive, quantitative analysis of the clinical, radiological, and CSF features of drug-associated PML and its subgroups, based on the underlying disease categories, has never been conducted thus far. Nevertheless, for an early diagnosis it is crucial for clinicians across various specialties to be aware of the preferential clinical and radiological presentation and the potential differences herein between the subgroups. In this study, we aimed to address this urgent medical need for advances in knowledge on drug-associated PML by outlining its specific clinical, radiological, and CSF characteristics. A quantitative assessment of the degree of association between any particular drug and PML has been reported previously, and therefore fell beyond the scope of this paper [7].

## Methods

### Inclusion of published reports

**Fig. 2:**
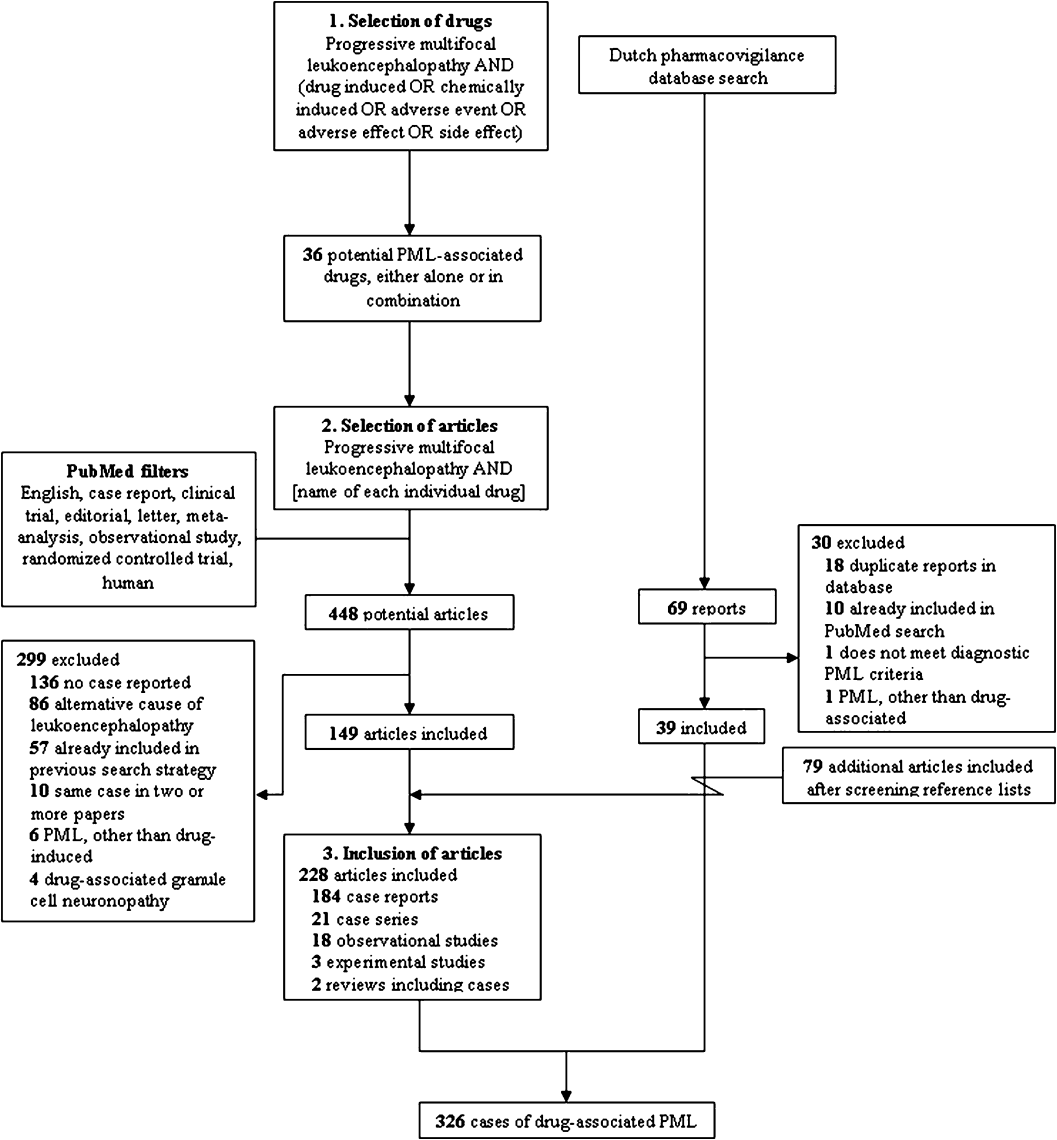
Flowchart of the search strategy, selection, and inclusion of articles and reports from the Dutch pharmacovigilance database

References were identified by means of a three-step PubMed search conducted on August 30, 2015 (Fig. 2). To obtain a first inventory of the drugs that might trigger PML occurrence, the search terms ‘progressive multifocal leukoencephalopathy’, ‘drug induced’, ‘chemically induced’, ‘medication induced’, ‘adverse event’, ‘adverse effect’, and ‘side effect’ were used in the ‘all fields’ menu without limits of time. Second, combinations of ‘progressive multifocal leukoencephalopathy’ and the names of each of the individual drugs that had been identified in the first step were applied to collect all reports of potentially drug-associated PML. Finally, reference lists of all included articles were screened for any additional relevant reports.

We included cases of possible, probable, and definite PML according to the current American Academy of Neurology criteria [3] that were published in English language journals in case reports, case series, clinical trials, editorials, letters, meta-analyses, and observational studies. In addition, articles were included when they described a simultaneous immune reconstitution inflammatory syndrome (IRIS), either occurring already during treatment with or only after withdrawal of the causative drug. With respect to the latter, IRIS was defined as deterioration of neurologic deficits following drug removal, corroborated by inflammatory changes on neuroimaging [8]. Articles were excluded if an identical case had already been described in a previous report, when PML was not related to a drug, or if an alternative diagnosis was more likely, e.g., posterior reversible encephalopathy syndrome (PRES) associated with tacrolimus, cyclosporine, cisplatin, and methotrexate. Finally, cases of medication-related granule cell neuronopathy were excluded [9, 10]. When imaging features played a pivotal role in the distinction between ‘possible PML’ and ‘not PML’, the decision of inclusion or exclusion was made after analysis by an experienced neuroradiologist with special expertise in the field of inflammatory diseases of the central nervous system (MPW).

### Dutch pharmacovigilance database

In addition to the cases published in the literature, reports on medication-associated PML were obtained by querying the database of the Netherlands Pharmacovigilance Centre Lareb. This database contains detailed accurately verified information of adverse drug reactions reported by Dutch healthcare professionals and manufacturers. Care was taken to exclude duplicates that had already been included in the literature search.

### Data acquisition

The following data, when available, were extracted from each report: study type, number of cases, age, sex, underlying disease, immunosuppressive agent(s), time elapsed between drug introduction and either first symptoms or asymptomatic radiological findings that could be attributed to PML in retrospect, mortality rate (and time from first symptoms to death), and clinical, radiological, CSF, and histopathological features. When a combination of therapies rather than a single pharmaceutical was presumed to be responsible for the phenotype, the most likely contributing drugs—based on mechanism of action, dose, and duration of use—were noted up to a maximum of three. The localization of PML lesions was determined by the description provided by the authors of each report and the MR and/or CT images shown, if any. In addition, the presence of contrast enhancement, its distribution pattern (i.e., patchy, punctate, homogeneous, or unknown), and the dissemination pattern of lesions were listed. With respect to the latter, we distinguished between unilobar, multilobar (i.e., two or more contiguous lobes affected), and widespread (i.e., two or more noncontiguous lobes affected and/or the presence of lesions in both hemispheres). For the sake of clinical relevance and vigilance, symptoms and radiological lesions that appeared after establishing the diagnosis of PML, attributable to either progression of the disease or a consecutive emerging IRIS, were excluded.

### Statistical analysis

Differences between the four subgroups, based on the underlying disease categories, were analyzed using Chi-square tests and one-way ANOVA. Post-hoc Bonferroni corrections have been applied and significance levels were set at 0.0083 (i.e., 0.05/6). Due to the conservative nature of the Bonferroni method [11], it seems likely that potential differences between the subgroups represent genuine dissimilarities rather than mere coincidence.

## Results

### Demographic and epidemiologic features

**Fig. 3:**
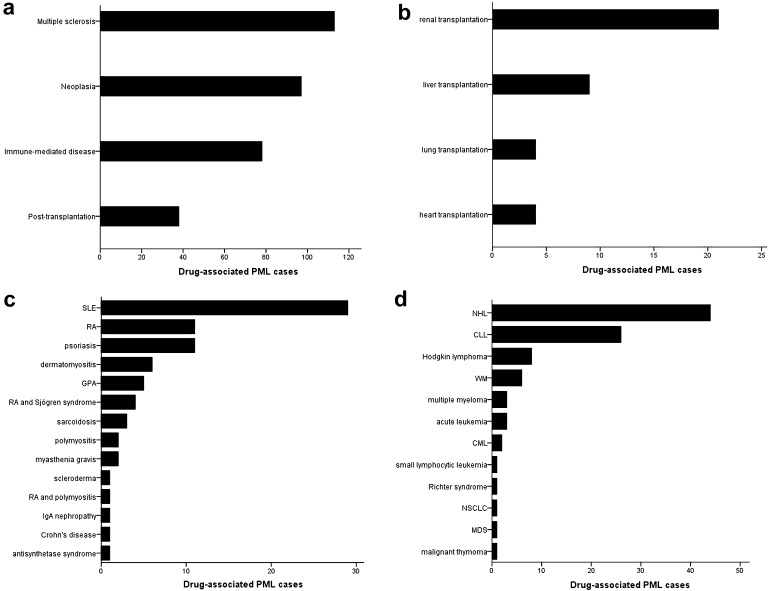
Distribution of cases of drug-associated progressive multifocal leukoencephalopathy among the major disease categories (**a**) and subdivisions of the post-transplantation setting (**b**), the autoimmune diseases (**c**), and the neoplasms (**d**). Because of the large number of natalizumab-associated PML cases, multiple sclerosis was considered a distinct entity and was not included in the group of autoimmune disorders. *CML* chronic myeloid leukemia, *CLL* chronic lymphocytic leukemia, *GPA* granulomatosis with polyangiitis (formerly Wegener’s disease), *MDS* myelodysplastic syndrome, *NHL* non-Hodgkin lymphoma, *NSCLC* non-small-cell lung cancer, *RA* rheumatoid arthritis, *SLE* systematic lupus erythematosus, *WM* Waldenström’s macroglobulinemia

The literature search yielded 287 cases of drug-related PML in 184 case reports, 21 case series, 18 observational studies, three experimental studies, and two review articles [12–239]. Furthermore, an additional 39 reports were identified in the Dutch pharmacovigilance database, resulting in a total number of 326 documented cases. In eight cases, consensus was reached after initial doubt on the diagnosis of PML; five of them have been included, while the remaining three were finally labeled as ‘not PML’. Natalizumab, predniso(lo)ne, (dimethyl) fumarate, fludarabine, rituximab, and brentuximab vedotin were the most common single agents that have been demonstrated to trigger PML (Online Resource 1). Additionally, glucocorticoids with either azathioprine or cyclophosphamide and a regime containing four or more pharmaceuticals [usually courses of chemotherapy, frequently including rituximab (R-CHOP)] comprised the most prevalent composite culprits. The multifarious underlying diseases could be subdivided into four main categories, i.e. multiple sclerosis (MS), (other) immune-mediated disorders, neoplasms (93.8 % lymphoproliferative, 3.1 % myeloproliferative, 2.1 % solid neoplasm, 1.0 % unknown), and the post-transplantation setting (Fig. 3). Because of the relatively large number of natalizumab-associated PML cases, the former was not included in the group of immune-mediated diseases, but rather considered a distinct entity. As immunosuppressive drugs are also frequently used in the prevention of relapses in neuromyelitis optica spectrum disorder after a first episode, one might expect drug-associated PML to have been described in this patient population. However, no such reports have been identified in our search. The most common drugs associated with PML per subgroup are summarized in Online Resource 2. In general, there was considerably few drug overlap between the various disease categories, an exception being the combination of azathioprine and glucocorticoids which was used in both autoimmune disorders and the post-transplantation setting.

The age and sex distribution of drug-related PML among the subgroups were in accordance with the epidemiologic features of the particular diseases, with a higher percentage of younger women and older men in the MS and neoplasm subgroups, respectively (Table 1). The mean time elapsed from introduction of the drug(s) to first symptoms was 28.9 (95 % CI 25.5–32.2) months, while the time span to the occurrence of asymptomatic lesions (only in natalizumab-induced PML) was found to be 36.4 (95 % CI 27.0–45.9) months. Significantly shorter periods were observed in the neoplasm group [mean 14.2 months (95 % CI 9.3–19.2)] compared to the three other disease categories (*p* < 0.001). A delay of several weeks to even months between the occurrence of first symptoms and the final diagnosis of PML was noticed in a considerable number of cases [23, 28, 50, 67, 78, 101, 103, 117, 122, 149, 158, 185, 201, 213, 223, 224, 226, 233, 235, 236]. This diagnosis was established on combined clinical, radiological, and CSF grounds in 69.4 % of cases. In the remaining 30.6 %, histopathological analysis was necessary to provide a greater degree of certainty, either because PCR for JCV in the CSF was not available or (repeatedly) negative. Brain biopsies were conducted most commonly in patients with underlying autoimmune diseases (43.8 %) and neoplasms (47.8 %), but in only 8.3 % of natalizumab-induced PML cases. The overall mortality rate was 52.2 % (95 % CI 46.5–58.0 %), with values of 12.0 % (95 % CI 5.2–18.7 %), 56.2 % (95 % CI 44.5–67.8 %), 83.3 % (95 % CI 75.5–91.2 %), and 68.4 % (95 % CI 52.9–83.9 %) in the MS, immune-mediated, neoplasm and post-transplantation subgroups, respectively.

**Table 1:** Demographic and epidemiologic features of drug-associated progressive multifocal leukoencephalopathy

	Multiple sclerosis^a^	Autoimmune diseases	Neoplasms	Post-transplantation	Total
Number of cases	113	78	97	38	326
Literature	89 (31.0)	75 (26.1)	86 (30.0)	37 (12.9)	287
Lareb	24 (61.5)	3 (7.7)	11 (28.2)	1 (2.6)	39
Age (years)	45.0 ± 10.4*^#^	54.5 ± 14.2*	58.9 ± 12.4^#¶^	49.7 ± 14.3^¶^	52.2 ± 13.7
Sex (% male)	41/111 (36.9)^¶†^	27/76 (35.5)*^#^	57/92 (62.0)*¶	25/38 (65.8)^#†^	150/317 (47.3)
Time to PML onset (months)	37.8 ± 17.0^#^	30.6 ± 20.5*	14.2 ± 21.4*^#¶^	34.9 ± 37.6^¶^	29.5 ± 24.4
To first symptoms	38.0 ± 17.5^#^	30.6 ± 20.5*	14.2 ± 21.4*^#¶^	34.9 ± 37.6^¶^	28.8 ± 25.3
To “silent” MRI lesions^b^	36.4 ± 12.2	N/A	N/A	N/A	36.4 ± 12.2
Diagnosis (%)					
Clinical–MRI–CSF	97/98 (99.0)*^#¶^	42/73 (57.5)*	52/93 (55.9)^#^	18/37 (48.6)^¶^	209/301 (69.4)
Histopathology	1/98 (1.0)*^#¶^	31/73 (42.5)*	41/93 (44.1)^#^	19/37 (51.4)^¶^	92/301 (30.6)
PML classification (%)					
Possible	11/99 (11.1)	4/76 (5.3)	9/94 (9.6)	3/38 (7.9)	27/307 (8.8)
Probable	12/99 (12.1)*^#¶^	0/76 (0.0)*	1/94 (1.1)^#^	0/38 (0.0)^¶^	13/307 (4.2)
Definite	75/99 (75.8)	63/76 (82.9)	79/94 (84.0)	28/38 (73.7)	245/307 (79.8)
Unknown	1/99 (1.0)*	9/76 (11.8)	5/94 (5.3)	7/38 (18.4)*	22/307 (7.2)
Mortality rate (%)	11/92 (12.0)*^#¶^	41/73 (56.2)*^†^	75/90 (83.3)^#†^	26/38 (68.4)^¶^	153/293 (52.2)
Time to death (months)	5.2 ± 3.2	7.0 ± 10.4	3.9 ± 4.5	3.4 ± 3.6	4.8 ± 6.7

Categorical data have been displayed as frequency (percentage), continuous data as mean ± standard deviation

^#^, *, ^¶^, ^†^ significant differences between subgroups (*p* < 0.05/6 = 0.0083)

^a^Because of the large number of natalizumab-associated PML cases, multiple sclerosis was considered a distinct entity and was not included in the group of autoimmune disorders

^b^Asymptomatic PML cases were only observed in natalizumab-treated patients

### Clinical characteristics

In descending order of magnitude, the most common overall symptoms and signs of drug-related PML were motor weakness (48.6 %), cognitive deficits (43.2 %), dysarthria (26.3 %), ataxia (24.1 %), aphasia (22.7 %), and behavioral changes (21.9 %) (Table 2). Although some deviations were observed between the subgroups, motor weakness and cognitive changes constituted the two most frequently encountered symptoms in all disease categories. Behavioral changes were significantly more common in the post-transplantation group than in any of the other disease categories (MS *p* < 0.001, immune-mediated disorders *p* = 0.001, neoplasms *p* = 0.002). The highest prevalences of cognitive deficits and seizures were also found in this disease category (60.0 and 22.9 %, respectively). Symptoms and signs of cerebellar and brainstem involvement, i.e., ataxia, vertigo, eye movement disorders, and dysphagia tended to be more prevalent in the group of autoimmune disorders. Furthermore, dysarthria, gait abnormalities and sensory deficits (37.3, 26.9, and 20.9 %, respectively) occurred most commonly in the group of immune-mediated diseases. A notable feature of drug-related PML among the neoplasms was the high prevalence of visual deficits (25.8 %), especially when compared to the autoimmune disorders (*p* = 0.001). Asymptomatic PML cases were only observed in natalizumab-treated patients (10.3 %).

**Table 2:** Clinical characteristics of drug-associated progressive multifocal leukoencephalopathy

	Multiple sclerosis (*n* = 87)	Autoimmune diseases (*n* = 67)	Neoplasms (*n* = 89)	Post-transplantation (*n* = 35)	Total (*n* = 278)
Motor weakness^a^	34 (39.1)	34 (50.7)	49 (55.1)	18 (51.4)	135 (48.6)
Cognitive deficits^b^	30 (34.5)	25 (37.3)	44 (49.4)	21 (60.0)	120 (43.2)
Dysarthria	13 (14.9)*	25 (37.3)*	27 (30.3)	8 (22.9)	73 (26.3)
Ataxia	14 (16.1)	21 (31.3)	23 (25.8)	9 (25.7)	67 (24.1)
Aphasia^c^	20 (23.0)	19 (28.4)	20 (22.5)	4 (11.4)	63 (22.7)
Behavioral change^d^	14 (16.1)^#^	12 (17.9)*	18 (20.2)^¶^	17 (48.6)^#^*^¶^	61 (21.9)
Gait abnormalities	14 (16.1)	18 (26.9)	13 (14.6)	5 (14.3)	50 (18.0)
Visual deficits^e^	10 (11.5)	4 (6.0)*	23 (25.8)*	6 (17.1)	43 (15.5)
Sensory deficits	7 (8.0)	14 (20.9)^#^	5 (5.6)^#^	6 (17.1)	32 (11.5)
Seizure	9 (10.3)	5 (7.5)	6 (6.7)	8 (22.9)	28 (10.1)
Facial palsy	5 (5.7)	10 (14.9)	6 (6.7)	6 (17.1)	27 (9.7)
Dysphagia	2 (2.3)	8 (11.9)	6 (6.7)	4 (11.4)	20 (7.2)
Apraxia	3 (3.4)	3 (4.5)	11 (12.4)	2 (5.7)	19 (6.8)
Vertigo	4 (4.6)	6 (9.0)	3 (3.4)	3 (8.6)	16 (5.8)
Eye movement disorders^f^	2 (2.3)	6 (9.0)	8 (9.0)	0 (0.0)	16 (5.8)
Headache	2 (2.3)	3 (4.5)	3 (3.4)	3 (8.6)	11 (4.0)
Parkinsonism^g^	1 (1.1)	6 (9.0)	2 (2.2)	2 (5.7)	11 (4.0)
Depression	4 (4.6)	2 (3.0)	1 (1.1)	1 (2.9)	8 (2.9)
Asymptomatic	9 (10.3)*^#^	0 (0.0)^#^	0 (0.0)*	0 (0.0)	9 (3.2)

Data have been displayed as frequency (percentage)

Because of the large number of natalizumab-associated PML cases, multiple sclerosis was considered a distinct entity and was not included in the group of autoimmune disorders

^a^Monoparesis, hemiparesis, tetraparesis, hemiplegia, and tetraplegia

^b^Confusion and memory deficits

^c^True aphasia and word finding difficulties

^d^Personality changes, apathy, lethargy, and agitation

^e^Visual field defects and reduced visual acuity

^f^Ophtalmoparesis, strabismus, and diplopia

^g^Tremor, bradykinesia, and ‘parkinsonism’

^#^, *, ^¶^ significant differences between subgroups (*p* < 0.05/6 = 0.0083)

### Radiological findings

Whereas infratentorial lesions were present in 27.4 % of cases, drug-associated PML was considerably more prevalent in the supratentorial brain (87.7 %) (Table 3). In all disease categories, lesions were most often situated in the frontal and parietal lobes (64.1 and 46.6 %, respectively). The temporal (21.4 %) and occipital (22.7 %) lobes were less commonly affected. Involvement of the latter, however, was observed relatively frequently in the neoplasm subgroup (36.2 %), in particular when compared to the autoimmune and MS groups (*p* = 0.003 and *p* = 0.01, respectively). Infratentorial lesion localization was especially prevalent in drug-related PML associated with immune-mediated disorders, with cerebellar involvement being significantly more common than in the MS and neoplasm categories (*p* = 0.001 and *p* = 0.007, respectively). Furthermore, there was a trend towards a higher level of brainstem involvement in the group of autoimmune diseases (MS *p* = 0.013; neoplasm *p* = 0.077). In the natalizumab-induced PML subgroup, lesions were most commonly distributed in one lobe (42.9 % vs. 21.4 % multilobar and 27.1 % widespread). In contrast, widespread dissemination was by far the most frequently encountered pattern in the remaining three disease categories (autoimmune disorders 52.5 %, neoplasms 52.1 %, post-transplantation 68.0 %). Contrast enhancement was present in 27.6 % of cases, particularly in the natalizumab-induced (42.9 %) and post-transplantation (31.6 %) subgroups. In the former, punctate and patchy enhancement patterns were observed to an equal extent (19.6 %). In the other subgroups, only patchy contrast enhancement was found.

**Table 3:** Radiological and cerebrospinal fluid features of drug-associated progressive multifocal leukoencephalopathy

	Multiple sclerosis^a^	Autoimmune diseases	Neoplasms	Post-transplantation	Total
Radiological features					
Supratentorial	66/72 (91.7)	46/61 (75.4)*	71/76 (93.4)*	24/27 (88.9)	207/236 (87.7)
Frontal lobe	49/69 (71.0)	32/57 (56.1)	43/69 (62.3)	17/25 (68.0)	141/220 (64.1)
Parietal lobe	22/69 (31.9)	31/58 (53.4)	36/69 (52.2)	14/25 (56.0)	103/221 (46.6)
Temporal lobe	9/69 (13.0)	12/57 (21.1)	21/69 (30.4)	5/25 (20.0)	47/220 (21.4)
Occipital lobe	10/69 (14.5)^#^	9/57 (15.8)	25/69 (36.2)^#^	6/25 (24.0)	50/220 (22.7)
Basal ganglia	1/69 (1.4)	3/57 (5.3)	3/69 (4.3)	3/25 (12.0)	10/220 (4.5)
Thalamus	3/69 (4.3)	6/58 (10.3)	8/69 (11.6)	3/25 (12.0)	20/221 (9.0)
Capsula interna	3/69 (4.3)	4/59 (6.8)	2/69 (2.9)	3/25 (12.0)	12/222 (5.4)
Corpus callosum	2/69 (2.9)	3/59 (5.1)	6/69 (8.7)	2/25 (8.0)	13/222 (5.9)
Infratentorial	13/69 (18.8)*	25/60 (41.7)*	15/69 (21.7)	8/25 (32.0)	61/223 (27.4)
Cerebellum	8/69 (11.6)*	21/60 (35.0)*^#^	10/69 (14.5)^#^	6/25 (24.0)	45/223 (20.2)
Brain stem	7/69 (10.1)	16/59 (27.1)	10/69 (14.5)	5/25 (20.0)	38/222 (17.1)
Lesion distribution					
Unilobar	30/70 (42.9)*^#^	13/59 (22.0)	13/71 (18.3)*	1/25 (4.0)^#^	57/225 (25.3)
Multilobar	15/70 (21.4)	13/59 (22.0)	17/71 (23.9)	6/25 (24.0)	51/225 (22.7)
Widespread	19/70 (27.1)*^#¶^	31/59 (52.5)*	37/71 (52.1)^#^	17/25 (68.0)^¶^	104/225 (46.2)
Unknown	6/70 (8.6)	2/59 (3.4)	4/71 (5.6)	1/25 (4.0)	13/225 (5.8)
Contrast enhancement	24/56 (42.9)*	5/43 (11.6)*	10/45 (22.2)	6/19 (31.6)	45/163 (27.6)
Punctate	11/56 (19.6)*^#^	0/43 (0.0)*	0/45 (0.0)^#^	0/19 (0.0)	11/163 (6.7)
Patchy	11/56 (19.6)	3/43 (7.0)	8/45 (17.8)	4/19 (21.1)	26/163 (16.0)
Homogeneous	1/56 (1.8)	0/43 (0.0)	0/45 (0.0)	0/19 (0.0)	1/163 (0.6)
Unknown	1/56 (1.8)	2/43 (4.7)	2/45 (4.4)	2/19 (10.5)	7/163 (4.3)
CSF features					
PCR JC virus					
Negative	11/95 (11.6)^#^	15/53 (28.3)	21/64 (32.8)^#^	6/21 (28.6)	53/233 (22.7)
Directly positive	64/95 (67.4)	31/53 (58.5)	39/64 (60.9)	14/21 (66.7)	148/233 (63.5)
Positive after ≥2 LPs	20/95 (21.1)*	7/53 (13.2)	4/64 (6.3)*	1/21 (4.8)	32/233 (13.7)
Leukocytes/ul					
0–4	17/19 (89.5)	38/41 (92.7)	34/41 (82.9)	17/18 (94.4)	106/119 (89.1)
≥5	2/19 (10.5)	3/41 (7.3)	7/41 (17.1)	1/18 (5.6)	13/119 (10.9)

Data have been displayed as frequency (percentage)

*LP* lumbar puncture

*, ^#^, ^¶^ significant differences between subgroups (*p* < 0.05/6 = 0.0083)

^a^Because of the large number of natalizumab-associated PML cases, multiple sclerosis was considered a distinct entity and was not included in the group of autoimmune disorders

### CSF results

JCV DNA remained (repeatedly) undetectable in 22.7 % (95 % CI 17.3–28.1 %) of drug-associated PML cases. Furthermore, nearly one out of each seven patients (13.7 %; 95 % CI 9.3–18.1 %) initially displayed one or more negative outcomes before PCR finally converted to positive, ranging from 4.8 % in the post-transplantation (95 % CI 0.0–14.7 %) subgroup to 21.1 % (95 % CI 12.7–29.4 %) in the MS subgroup. Positive results in the first CSF sample were obtained in 63.5 %. Pleocytosis was present in 10.9 % of patients, especially in the neoplasm group (17.1 %).

## Discussion

Despite the exponential increase in the number of drug-associated PML cases throughout the years, a comprehensive, quantitative analysis of its diagnostic characteristics that also takes into account the differences between the various underlying disease categories has not been performed thus far. Our study demonstrates that motor weakness and cognitive deficits are the most common presenting symptoms in all subgroups. Several dissimilarities were observed, however, between the disease categories in the remaining clinical manifestations. While behavioral and cognitive changes were most prevalent among the post-transplantation group, cerebellar and brainstem symptoms occurred most commonly in the autoimmune category. Visual disturbances appeared most frequently in drug-related PML associated with neoplasms. However, a sound explanation for these differences seems to be missing.

Because of the frequently grim prognosis and lethal outcomes, it is of paramount importance to consider the diagnosis of medication-associated PML at an early stage during treatment with immunosuppressive drugs. The clinical phenotype in the articles reviewed, however, was not seldom initially misinterpreted as an exacerbation or the cerebral manifestations of the underlying disease, i.e., an MS relapse, neuropsychiatric lupus, or central nervous system vasculitis [23, 28, 50, 67, 78, 101, 103, 117, 122, 149, 158, 185, 201, 213, 223, 224, 226, 233, 235, 236]. It was only after further clinical and radiological worsening upon high doses of immunosuppressive therapies that the possibility of PML was considered in these cases. Apart from the intrinsically dismal prognosis of PML, this delay of several weeks to even months and additional assault on the immune system certainly may have contributed to the high mortality rate. Sometimes, acute symptoms and signs were attributed to cerebral infarctions [21, 38, 109]. Finally, ocular and vestibular symptoms have occasionally been misjudged as cataract and Meniere’s disease, respectively [45, 120, 155]. It is possible that PML symptoms have been misinterpreted in these cases because of the relatively long interval between drug introduction and first central nervous system deficits, on average almost 2.5 years.

The differential diagnosis of medication-associated PML depends on the context in which the specific immunosuppressive drug is applied. In the post-transplantation setting, it is important to distinguish between drug-associated PML and PRES, which usually affects the parieto-occipital lobes and frequently resolves spontaneously after withdrawal of the culprit(s). In MS patients, on the other hand, the distinction between a relapse and natalizumab-induced PML can be particularly troublesome. A correct and early diagnosis, however, has important therapeutic and prognostic implications. In this regard, several clinical and MRI features may direct the physician to the right diagnosis. Acute spinal cord or brainstem presentations and focal, sharp-edged, periventricular locations are generally more frequently encountered in an MS exacerbation. In contrast, PML is characterized by a subacute onset, progressive nature of (sub)cortical symptoms, and large, ill-defined, confluent T2-weighted lesions, deep gray matter involvement, and/or crescent cerebellar lesions [240, 241]. This study confirms these findings, the most common natalizumab-associated PML symptoms being motor weakness, cognitive deficits, and aphasia, and lesions mainly localized in the frontal and parietal lobes. Isolated brainstem involvement, however, does not exclude PML at all [201, 233]. New imaging procedures such as the application of susceptibility weighted imaging and ultra high-field 7 Tesla MRI have shown promising preliminary data in the distinction between PML and MS lesions and might finally play a pivotal role when regular techniques fail to attain full assurance [242]. However, further work is required before they can be implemented in everyday clinical practice.

Due to the raised level of radiological vigilance in natalizumab-treated patients including high-frequency MRI monitoring according to recent expert opinion guidelines [243, 244], cases of PML diagnosed at an asymptomatic stage are identified with increasing frequency [19, 27, 123, 137, 153, 162, 221]. These cases are only classified as probable PML in the presence of JCV DNA in the CSF detected by PCR, indicating the conservative nature of the current diagnostic criteria [3]. The predicament of negative or inconclusive results across different laboratories in view of asymptomatic patients displaying MRI lesions has been touched upon previously [221] and stresses the need of a revision of the diagnostic PML criteria.

Definitive establishment of the PML diagnosis was hampered by initially negative PCR outcomes in CSF samples in nearly one out of each seven patients before it finally converted to positive. These results are strongly dependent on the disease stage and the characteristics of the assay, especially its lower detection limit, the sensitivity of the older assays being approximately 75 % [3]. Samples were frequently analyzed not only in the institute’s own laboratory, but also in external facilities, with the National Institutes of Health laboratory being the one with the most ultrasensitive assay (i.e., a detection limit of 10 JCV DNA copies per milliliter). Thus, physicians need to be aware that the lack of detection of JCV DNA in CSF does not preclude the diagnosis of PML. Repeated lumbar punctures, follow-up MRI, and possibly additional techniques currently under evaluation such as the assessment of anti-JCV antibodies in CSF [245], and in a proportion of cases, biopsy may be required to confirm the diagnosis of PML.

A clear radiological anterior-posterior gradient was observed, with the frontal and parietal lobes being more often involved than their temporal and occipital counterparts. A widespread lesion distribution pattern was found to be most common in drug-related PML associated with autoimmune disorders, neoplasms, and the post-transplantation setting, while (asymptomatic) unilobar involvement was most often observed in the natalizumab-induced subgroup. Contrast enhancement was present in about one fourth of cases of drug-associated PML, especially in the MS and post-transplantation subgroups. Although the underlying mechanisms and evolution of this ‘inflammatory PML’ variant remain to be elucidated, experimental evidence in natalizumab-induced PML suggests that lymphocyte trafficking continues to occur via alternative pathways due to upregulation of different adhesion molecules, thereby inducing a state of incomplete immune surveillance [246].

In contrast to what its name may suggest, the thalamus and basal ganglia—obviously deep gray matter structures—were involved in the PML disease process in 9.0 and 4.5 % of cases, respectively. Berger et al. previously demonstrated lesions in these areas in 14 and 12 % in PML associated with human immunodeficiency virus [247]. Furthermore, seizures which are generally assumed to be generated by synchronous cortical activity were present in 10.1 % of patients. In previous studies, even higher prevalences of 18 and 34.7 % were found, with causative PML lesions being situated adjacent to the T1-hyperintense cerebral cortical gray matter [248, 249]. It can be inferred from both observations that the ‘leukoencephalopathy’ phrase in the term PML, suggesting a white matter disease, is actually kind of a misnomer and rather confusing for clinicians who are not familiar with this disease.

The discrepancies in survival between the different categories of PML—not only the drug-associated cases, but also the ones related to an intrinsically compromised immune system—can probably be explained by their various degrees of ‘generalized’ immunosuppression. The more specific the immune system is targeted, the lower the mortality rate in general. We demonstrated a significantly lower mortality rate in the natalizumab-induced PML subgroup compared to the other disease categories and found the highest value in the neoplasm subgroup. As natalizumab impedes the interaction between alpha-4 integrin on lymphocytes and vascular cell adhesion molecule 1 on the endothelium of the vessel wall, it specifically prevents the diapedesis of these cells through the blood brain barrier without reducing the total number of leukocytes in the blood or their function. Therefore, in contrast to the various antineoplastic agents and immunosuppressive drugs used in cancer and the post-transplantation setting, the immune system may be more specifically suppressed. Depression of the immune system may not only result from the drugs administered, but is also an intrinsic consequence of the disease process in hematological malignancies which accounts for the highest mortality rate in this subgroup. Furthermore, the high level of radiological vigilance and occurrence of asymptomatic PML cases among MS patients treated with natalizumab frequently led to a quick drug suspension which probably contributed to a better prognosis.

Several intrinsic limitations of this study need to be addressed. Although we identified a large number of reports, not all cases of medication-related PML are published in the literature, especially when the association between the drug and the side effect has been described before. Therefore, our study design does not allow calculating the treatment-related risk of developing PML for each specific drug. Second, the contrast between underreporting in the oncology field on the one hand and the high clinical and radiological vigilance in MS and autoimmune disorders on the other has possibly led to a skewed distribution of published cases. Furthermore, the causal relationship between the mentioned drugs and PML may have been blurred by a variety of reasons. First, a state of immunosuppression could have already been present as a result of the underlying disease itself, e.g., due to lymphopenia in SLE, sarcoidosis, or leukemia. Indeed, PML has been described in these disorders in the absence of immunosuppressive medication [250, 251]. Therefore, it seems plausible to assume that PML in the group of neoplasms and in some but not all autoimmune disorders results from the interaction and synergistic immunosuppressive effects of the given drug(s) and the underlying disease. In the MS and post-transplantation setting, on the other hand, PML seems to be entirely attributable to iatrogenic reasons. Second, it is not inconceivable that several reports only mentioned the drug(s) that were taken at the time of PML onset, without giving notice to previous use of immunosuppressive therapies or long-standing leukopenia. As a result, the drugs mentioned may just have given the final push to the diagnosis of PML rather than being fully responsible. In most cases of this study (277/326, 85 %), the risk of medication-associated PML seemed related to a maximum of three drugs. However, in this respect the heterogeneity of the data set needs to be appreciated and one should take into account several other factors like duration of treatment, concomitant medication use, and comorbidity.

In conclusion, drug-associated PML represents an emerging yet underrecognized clinical entity. We demonstrated that the various subgroups share several clinical and radiological characteristics despite the highly heterogeneous nature of the underlying diseases. In all subgroups, motor weakness and cognitive changes comprised the two most common clinical manifestations, while the frontal and parietal lobes invariably appeared to be the predilection sites of PML lesions. Nonetheless, it is important to be aware of subtle—and sometimes more obvious—differences in preferential presentations, e.g., discrepancies in the patterns of lesion dissemination and contrast enhancement. In the end, meticulous clinical and radiological vigilance in recognizing the phenotype at an early stage coupled with prompt withdrawal and clearance of the culprit, appropriate supportive therapies, and accurate monitoring and treatment of a consecutive IRIS remain crucial to obtain better outcomes in the battle against this frequently relentless disease.

## Electronic supplementary material

Below is the link to the electronic supplementary material.

Supplementary material 1 (DOCX 299 kb)
