# Supplementary material for: Drug-associated progressive multifocal leukoencephalopathy: a clinical, radiological, and cerebrospinal fluid analysis of 326 cases
Source: J Neurol. 2016 Jul 11;263(10):2004–21. doi: 10.1007/s00415-016-8217-x (PMC5037162; doi:10.1007/s00415-016-8217-x)
Supplement: Supplementary file 1 — Supplementary material 1 (DOCX 299 kb) [file 415_2016_8217_MOESM1_ESM.docx]

**Drug-associated progressive multifocal leukoencephalopathy**

*A clinical, radiological, and cerebrospinal fluid analysis of 326 cases*

Roderick P.P.W.M. Maas, Annemarie H.G. Muller-Hansma, Rianne A.J. Esselink, Jean-Luc Murk, Clemens Warnke, Joep Killestein, Mike P. Wattjes

**Corresponding author**

Roderick Maas, MD

Department of Neurology, route 935, Radboud University Medical Center

PO Box 9101, 6500 HB Nijmegen, The Netherlands

(e) [roderick.maas@radboudumc.nl](mailto:roderick.maas@radboudumc.nl)

**Journal**

Journal of Neurology

**Online Resource 1**. List of (combinations of) drugs that have been shown to be associated with progressive multifocal leukoencephalopathy.

|  | **All** | **PubMed** | **Lareb** |  |  | **All** | **PubMed** | **Lareb** |
| --- | --- | --- | --- | --- | --- | --- | --- | --- |
| **Natalizumab** | 113 | 89 | 24 |  | **Rituximab + GC** | 2 | 1 | 1 |
| **≥ 4 drugs** | 49 | 42 | 7 |  | **MTX + rituximab** | 2 | 2 | 0 |
| **AZA + GC** | 15 | 15 | 0 |  | **Adalimumab** | 1 | 1 | 0 |
| **Predniso(lo)ne** | 14 | 14 | 0 |  | **Alemtuzumab + GC** | 1 | 1 | 0 |
| **CYC + GC** | 12 | 11 | 1 |  | **Alemtuzumab + GC+ TAC** | 1 | 1 | 0 |
| **(Dimethyl) fumarate** | 9 | 9 | 0 |  | **Azathioprine** | 1 | 1 | 0 |
| **Fludarabine** | 8 | 8 | 0 |  | **MMF + belatacept** | 1 | 1 | 0 |
| **Rituximab** | 7 | 6 | 1 |  | **Bendamustine + rituximab** | 1 | 1 | 0 |
| **Brentuximab vedotin** | 6 | 5 | 1 |  | **Oxaliplatin + capecitabine** | 1 | 1 | 0 |
| **CYC + FDR + rituximab** | 6 | 6 | 0 |  | **Carboplatin + gemcitabine** | 1 | 1 | 0 |
| **CsA + AZA + GC** | 5 | 5 | 0 |  | **Cladribine + mitoxantrone** | 1 | 1 | 0 |
| **MMF + GC** | 5 | 5 | 0 |  | **CsA + CYC + GC** | 1 | 1 | 0 |
| **Cyclophosphamide** | 4 | 4 | 0 |  | **Dapsone + GC** | 1 | 1 | 0 |
| **CsA + GC** | 4 | 4 | 0 |  | **Etanercept + GC** | 1 | 1 | 0 |
| **MTX + GC** | 4 | 4 | 0 |  | **FDR + CYC** | 1 | 1 | 0 |
| **Alemtuzumab** | 3 | 2 | 1 |  | **Infliximab + MTX + GC** | 1 | 1 | 0 |
| **Chlorambucil** | 3 | 3 | 0 |  | **MMF + GC + rapamycine** | 1 | 1 | 0 |
| **CsA + MMF + GC** | 3 | 3 | 0 |  | **MMF + TAC** | 1 | 1 | 0 |
| **Efalizumab** | 3 | 3 | 0 |  | **Rituximab + CsA** | 1 | 1 | 0 |
| **AZA + CYC + GC** | 2 | 2 | 0 |  | **Rituximab + FDR + GC** | 1 | 1 | 0 |
| **Belimumab + MMF + GC** | 2 | 2 | 0 |  | **Rituximab + MTX + CsA** | 1 | 1 | 0 |
| **CYC + etoposide** | 2 | 2 | 0 |  | **Rituximab + leflunomide** | 1 | 1 | 0 |
| **FDR + mitoxantrone + CYC** | 2 | 2 | 0 |  | **AZA + GC + TAC** | 1 | 1 | 0 |
| **FDR + rituximab** | 2 | 2 | 0 |  | **Tocilizumab** | 1 | 1 | 0 |
| **Leflunomide** | 2 | 2 | 0 |  | **MTX + CsA** | 1 | 1 | 0 |
| **Methotrexate** | 2 | 2 | 0 |  | **CYC + CsA + FDR** | 1 | 1 | 0 |
| **Mycophenolate mofetil** | 2 | 1 | 1 |  | **Rituximab + MTX + GC** | 1 | 0 | 1 |
| **MMF + TAC + GC** | 2 | 2 | 0 |  | **CYC + GC + lenalidomide** | 1 | 0 | 1 |
| **TAC + GC** | 2 | 2 | 0 |  | **Chlorambucil + GC** | 1 | 1 | 0 |
| **CYC + rituximab** | 2 | 2 | 0 |  |  |  |  |  |

AZA = azathioprine, GC = glucocorticoids, CYC = cyclophosphamide, FDR = fludarabine, CsA = cyclosporine, MMF = mycophenolate mofetil, MTX = methotrexate, TAC = tacrolimus.

**Online Resource 2.** Most common drugs that have been shown to be associated with PML in the various subgroups (n ≥ 5 reports).

| **Multiple sclerosis**  **(n = 113)^a^** | **Autoimmune diseases**  **(n = 78)** | **Neoplasms**  **(n = 97)** | **Post-transplantation**  **(n = 38)** |
| --- | --- | --- | --- |
| Natalizumab  (112; 99%) | Predniso(lo)ne  (14; 17.9%)  AZA + GC  (8; 10.3%)  Fumarate  (8; 10.3%)  CYC + GC  (7; 9.0%) | ≥ 4 drugs  (42; 43.3%)  Fludarabine  (8; 8.2%)  Brentuximab vedotin (6; 6.2%)  CYC + RIT + FDR  (6; 6.2%)  Rituximab  (5; 5.2%) | AZA + GC  (7; 18.4%)  CsA + GC + AZA  (5; 13.2%) |

^a^ Because of the large number of natalizumab-associated PML cases, multiple sclerosis was considered a distinct entity and was not included in the group of autoimmune disorders.

AZA = azathioprine, GC = glucocorticoids, CYC = cyclophosphamide, FDR = fludarabine, CsA = cyclosporine, RIT = rituximab.

**Combinations of drugs and cited papers (for order of references see below)**

Natalizumab: [1-58]

≥ 4 drugs: [18, 59-95]

Azathioprine + glucocorticoids: [96-110]

Predniso(lo)ne: [102, 111-122]

Cyclophosphamide + glucocorticoids: [106, 123-131]

(Dimethyl) fumarate: [132-140]

Fludarabine: [94, 141-145]

Rituximab: [18, 85, 146-149]

Brentuximab vedotin: [150-152]

Cyclophosphamide + fludarabine + rituximab: [153-158]

Cyclosporine + azathioprine + glucocorticoids: [159-163]

Mycophenolate mofetil + glucocorticoids: [126, 164-167]

Cyclophosphamide: [106, 168-170]

Cyclosporine + glucocorticoids: [171-174]

Methotrexate + glucocorticoids: [175-178]

Alemtuzumab: [179, 180]

Chlorambucil: [106, 181, 182]

Cyclosporine + mycophenolate mofetil + glucocorticoids: [122, 183, 184]

Efalizumab: [185]

Azathioprine + cyclophosphamide + glucocorticoids: [186, 187]

Belimumab + mycophenolate mofetil + glucocorticoids: [188, 189]

Cyclophosphamide + etoposide: [190, 191]

Fludarabine + mitoxantrone + cyclophosphamide: [192, 193]

Fludarabine + rituximab: [153, 194]

Leflunomide: [195, 196]

Methotrexate: [197, 198]

Mycophenolate mofetil: [199]

Mycophenolate mofetil + tacrolimus + glucocorticoids: [200, 201]

Tacrolimus + glucocorticoids: [202, 203]

Cyclophosphamide + rituximab: [153, 204]

Rituximab + glucocorticoids: [205]

Methotrexate + rituximab: [146]

Adalimumab: [206]

Alemtuzumab + glucocorticoids: [207]

Alemtuzumab + glucocorticoids + tacrolimus: [208]

Azathioprine: [209]

Mycophenolate mofetil + belatacept: [210]

Bendamustine + rituximab: [211]

Oxaliplatin + capecitabine: [212]

Carboplatin + gemcitabine: [213]

Cladribine + mitoxantrone: [214]

Cyclosporine + cyclophosphamide + glucocorticoids: [215]

Dapsone + glucocorticoids: [216]

Etanercept + glucocorticoids: [217]

Fludarabine + cyclophosphamide: [218]

Infliximab + methotrexate + glucocorticoids: [219]

Mycophenolate mofetil + glucocorticoids + rapamycine: [220]

Mycophenolate mofetil + tacrolimus: [221]

Rituximab + cyclosporine: [222]

Rituximab + fludarabine + glucocorticoids: [223]

Rituximab + methotrexate + cyclosporine: [93]

Rixumab + leflunomide: [146]

Azathioprine + glucocorticoids + tacrolimus: [224]

Tocilizumab: [225]

Methotrexate + cyclosporine: [226]

Cyclophosphamide + cyclosporine + fludarabine: [227]

Chlorambucil + glucocorticoids: [228]

**References (order of references according to the list above)**

1. Aksamit AJ, Jr. (2012) Progressive multifocal leukoencephalopathy. Continuum (Minneap Minn ) 18:1374-1391.

2. Arnaud FX, Hissene A, Metivier D, Dutasta F, Berets O, N'guema B, A'teriitehau C, Baccialone J, Potet J (2012) Gadolinium enhancement in brain magnetic resonance imaging in progressive multifocal leukoencephalopathy after natalizumab monotherapy: is it really atypical? J Neuroradiol 39:267-270.

3. Ayzenberg I, Lukas C, Trampe N, Gold R, Hellwig K (2012) Value of MRI as a surrogate marker for PML in natalizumab long-term therapy. J Neurol 259:1732-1733.

4. Berghoff M, Dassinger B, Iwinska-Zelder J, Giraldo M, Bilgin S, Kaps M, Gizewski ER (2014) A case of natalizumab-associated progressive multifocal leukoencephalopathy-role for advanced MRI? Clin Neuroradiol 24:173-176.

5. Blair NF, Brew BJ, Halpern JP (2012) Natalizumab-associated PML identified in the presymptomatic phase using MRI surveillance. Neurology 78:507-508.

6. Blinkenberg M, Sellebjerg F, Leffers AM, Madsen CG, Sorensen PS (2013) Clinically silent PML and prolonged immune reconstitution inflammatory syndrome in a patient with multiple sclerosis treated with natalizumab. Mult Scler 19:1226-1229.

7. Boster AL, Nicholas JA, Topalli I, Kisanuki YY, Pei W, Morgan-Followell B, Kirsch CF, Racke MK, Pitt D (2013) Lessons learned from fatal progressive multifocal leukoencephalopathy in a patient with multiple sclerosis treated with natalizumab. JAMA Neurol 70:398-402.

8. Calvi A, De RM, Pietroboni AM, Ghezzi L, Maltese V, Arighi A, Fumagalli GG, Jacini F, Donelli C, Comi G, Galimberti D, Scarpini E (2014) Partial recovery after severe immune reconstitution inflammatory syndrome in a multiple sclerosis patient with progressive multifocal leukoencephalopathy. Immunotherapy 6:23-28.

9. Clerico M, Schiavetti I, De Mercanti SF, Piazza F, Gned D, Brescia M, V, Lanzillo R, Ghezzi A, Bianchi A, Salemi G, Realmuto S, Sola P, Vitetta F, Cavalla P, Paolicelli D, Trojano M, Sormani MP, Durelli L (2014) Treatment of relapsing-remitting multiple sclerosis after 24 doses of natalizumab: evidence from an Italian spontaneous, prospective, and observational study (the TY-STOP Study). JAMA Neurol 71:954-960.

10. Damasceno A, von Glehn F, Martinez AR, Longhini AL, Deus-Silva L, Brandao CO, Santos LM, Damasceno BP (2011) Early onset of natalizumab-related progressive multifocal leukoencephalopathy. Mult Scler 17:1397-1398.

11. Dominguez-Mozo MI, Garcia-Montojo M, De LH, V, Garcia-Martinez A, Arias-Leal AM, Casanova I, Arroyo R, Alvarez-Lafuente R (2013) Anti-JCV antibodies detection and JCV DNA levels in PBMC, serum and urine in a cohort of Spanish Multiple Sclerosis patients treated with natalizumab. J Neuroimmune Pharmacol 8:1277-1286.

12. Eisele P, Szabo K, Hornberger E, Griebe M, Hennerici MG, Kieseier BC, Gass A (2014) Presumptive progressive multifocal leukoencephalopathy in multiple sclerosis after natalizumab therapy. J Neuroimaging 24:425-428.

13. Elster MJ (2013) Natalizumab (tysabri)-associated progressive multifocal leukoencephalopathy: insights from perfusion magnetic resonance imaging. J Comput Assist Tomogr 37:694-697.

14. Fine AJ, Sorbello A, Kortepeter C, Scarazzini L (2014) Progressive multifocal leukoencephalopathy after natalizumab discontinuation. Ann Neurol 75:108-115.

15. Gajofatto A, Bianchi MR, Deotto L, Benedetti MD (2014) Are natalizumab and fingolimod analogous second-line options for the treatment of relapsing-remitting multiple sclerosis? A clinical practice observational study. Eur Neurol 72:173-180.

16. Gheuens S, Smith DR, Wang X, Alsop DC, Lenkinski RE, Koralnik IJ (2012) Simultaneous PML-IRIS after discontinuation of natalizumab in a patient with MS. Neurology 78:1390-1393.

17. Giacomini PS, Rozenberg A, Metz I, Araujo D, Arbour N, Bar-Or A (2014) Maraviroc and JC virus-associated immune reconstitution inflammatory syndrome. N Engl J Med 370:486-488.

18. Haghikia A, Perrech M, Pula B, Ruhrmann S, Potthoff A, Brockmeyer NH, Goelz S, Wiendl H, Linda H, Ziemssen T, Baranzini SE, Kall TB, Bengel D, Olsson T, Gold R, Chan A (2011) Functional energetics of CD4+-cellular immunity in monoclonal antibody-associated progressive multifocal leukoencephalopathy in autoimmune disorders. PLoS One 6:e18506.

19. Havla J, Berthele A, Kumpfel T, Krumbholz M, Jochim A, Kronsbein H, Ryschkewitsch C, Jensen P, Lippmann K, Hemmer B, Major E, Hohlfeld R (2013) Co-occurrence of two cases of progressive multifocal leukoencephalopathy in a natalizumab "infusion group". Mult Scler 19:1213-1215.

20. Havla J, Hohlfeld R, Kumpfel T (2014) Unusual natalizumab-associated progressive multifocal leukoencephalopathy starting in the brainstem. J Neurol 261:232-234.

21. Hendel-Chavez H, de Goer de Herve MG, Giannesini C, Mazet AA, Papeix C, Louapre C, Chardain A, Boutarfa N, Theaudin M, Adams D, Gasnault J, Stankoff B, Taoufik Y (2013) Immunological hallmarks of JC virus replication in multiple sclerosis patients on long-term natalizumab therapy. J Virol 87:6055-6059.

22. Holmen C, Piehl F, Hillert J, Fogdell-Hahn A, Lundkvist M, Karlberg E, Nilsson P, Dahle C, Feltelius N, Svenningsson A, Lycke J, Olsson T (2011) A Swedish national post-marketing surveillance study of natalizumab treatment in multiple sclerosis. Mult Scler 17:708-719.

23. Kleinschmidt-DeMasters BK, Tyler KL (2005) Progressive multifocal leukoencephalopathy complicating treatment with natalizumab and interferon beta-1a for multiple sclerosis. N Engl J Med 353:369-374.

24. Kleinschmidt-DeMasters BK, Miravalle A, Schowinsky J, Corboy J, Vollmer T (2012) Update on PML and PML-IRIS occurring in multiple sclerosis patients treated with natalizumab. J Neuropathol Exp Neurol 71:604-617.

25. Kleiter I, Schroder M, Lurding R, Schuierer G, Clifford DB, Bogdahn U, Steinbrecher A, Poschl P (2010) Early changes on electroencephalography in natalizumab-associated progressive multifocal leucoencephalopathy. Mult Scler 16:749-753.

26. Ko MY, Stefoski D, Balabanov R (2011) Indolent course of progressive multifocal leukoencephalopathy during natalizumab treatment in MS. Neurology 77:1020.

27. Kuhle J, Gosert R, Buhler R, Derfuss T, Sutter R, Yaldizli O, Radue EW, Ryschkewitsch C, Major EO, Kappos L, Frank S, Hirsch HH (2011) Management and outcome of CSF-JC virus PCR-negative PML in a natalizumab-treated patient with MS. Neurology 77:2010-2016.

28. Lalive PH, Bridel C, Ferfoglia RI, Kaiser L, Du PR, Barkhof F, Haller S (2015) Minimal supportive treatment in natalizumab-related PML in a MS patient. J Neurol Neurosurg Psychiatry 86:354-355.

29. Langer-Gould A, Atlas SW, Green AJ, Bollen AW, Pelletier D (2005) Progressive multifocal leukoencephalopathy in a patient treated with natalizumab. N Engl J Med 353:375-381.

30. Lee DH, Waschbisch A, Lammer AB, Doerfler A, Schwab S, Linker RA (2013) Immunological and clinical consequences of splenectomy in a multiple sclerosis patient treated with natalizumab. J Neuroinflammation 10:123.

31. Linda H, von HA, Major EO, Ryschkewitsch C, Berg J, Olsson T, Martin C (2009) Progressive multifocal leukoencephalopathy after natalizumab monotherapy. N Engl J Med 361:1081-1087.

32. Linda H, von HA (2013) Presymptomatic diagnosis with MRI and adequate treatment ameliorate the outcome after natalizumab-associated progressive multifocal leukoencephalopathy. Front Neurol 4:11.

33. Maillart E, Louapre C, Lubetzki C, Papeix C (2014) Fingolimod to treat severe multiple sclerosis after natalizumab-associated progressive multifocal leukoencephalopathy: a valid option? Mult Scler 20:505-509.

34. Mazda ME, Brosch JR, Wiens AL, Bonnin JM, Kamer AP, Mattson DH, Snook RJ (2013) A case of natalizumab-associated progressive multifocal leukoencephalopathy with repeated negative CSF JCV testing. Int J Neurosci 123:353-357.

35. Mc Govern EM, Hennessy MJ (2013) Asymptomatic progressive multifocal leukoencephalopathy associated with natalizumab. J Neurol 260:665-667.

36. Metivier D, Arnaud FX, Dutasta F, Nguema B, Teriitehau C, Berets O, Baccialone J, Potet J (2013) Immune reconstitution inflammatory syndrome in a patient treated with natalizumab presenting progressive multifocal leukoencephalopathy. Diagn Interv Imaging 94:101-103.

37. Metz I, Radue EW, Oterino A, Kumpfel T, Wiendl H, Schippling S, Kuhle J, Sahraian MA, Gray F, Jakl V, Hausler D, Bruck W (2012) Pathology of immune reconstitution inflammatory syndrome in multiple sclerosis with natalizumab-associated progressive multifocal leukoencephalopathy. Acta Neuropathol 123:235-245.

38. Outteryck O, Ongagna JC, Duhamel A, Zephir H, Collongues N, Lacour A, Fleury MC, Berteloot AS, Blanc F, Giroux M, Vermersch P, de SJ (2012) Anti-JCV antibody prevalence in a French cohort of MS patients under natalizumab therapy. J Neurol 259:2293-2298.

39. Outteryck O, Ongagna JC, Brochet B, Rumbach L, Lebrun-Frenay C, Debouverie M, Zephir H, Ouallet JC, Berger E, Cohen M, Pittion S, Laplaud D, Wiertlewski S, Cabre P, Pelletier J, Rico A, Defer G, Derache N, Camu W, Thouvenot E, Moreau T, Fromont A, Tourbah A, Labauge P, Castelnovo G, Clavelou P, Casez O, Hautecoeur P, Papeix C, Lubetzki C, Fontaine B, Couturier N, Bohossian N, Clanet M, Vermersch P, de SJ, Brassat D (2014) A prospective observational post-marketing study of natalizumab-treated multiple sclerosis patients: clinical, radiological and biological features and adverse events. The BIONAT cohort. Eur J Neurol 21:40-48.

40. Phan-Ba R, Lommers E, Tshibanda L, Calay P, Dubois B, Moonen G, Clifford D, Belachew S (2012) MRI preclinical detection and asymptomatic course of a progressive multifocal leucoencephalopathy (PML) under natalizumab therapy. J Neurol Neurosurg Psychiatry 83:224-226.

41. Piehl F, Holmen C, Hillert J, Olsson T (2011) Swedish natalizumab (Tysabri) multiple sclerosis surveillance study. Neurol Sci 31 Suppl 3:289-293.

42. Piola M, Di PF, Mascoli N, Binda S, Arnaboldi M, Rezzonico M (2014) Atypical MRI features at early onset natalizumab-associated progressive multifocal leukoencephalopathy: a case report. J Neurol Sci 340:213-214.

43. Schroder A, Lee DH, Hellwig K, Lukas C, Linker RA, Gold R (2010) Successful management of natalizumab-associated progressive multifocal leukoencephalopathy and immune reconstitution syndrome in a patient with multiple sclerosis. Arch Neurol 67:1391-1394.

44. Schwab N, Hohn KG, Schneider-Hohendorf T, Metz I, Stenner MP, Jilek S, Du Pasquier RA, Gold R, Meuth SG, Ransohoff RM, Bruck W, Wiendl H (2012) Immunological and clinical consequences of treating a patient with natalizumab. Mult Scler 18:335-344.

45. Schwab N, Schneider-Hohendorf T, Posevitz V, Breuer J, Gobel K, Windhagen S, Brochet B, Vermersch P, Lebrun-Frenay C, Posevitz-Fejfar A, Capra R, Imberti L, Straeten V, Haas J, Wildemann B, Havla J, Kumpfel T, Meinl I, Niessen K, Goelz S, Kleinschnitz C, Warnke C, Buck D, Gold R, Kieseier BC, Meuth SG, Foley J, Chan A, Brassat D, Wiendl H (2013) L-selectin is a possible biomarker for individual PML risk in natalizumab-treated MS patients. Neurology 81:865-871.

46. Sottini A, Capra R, Zanotti C, Chiarini M, Serana F, Ricotta D, Caimi L, Imberti L (2012) Pre-existing T- and B-cell defects in one progressive multifocal leukoencephalopathy patient. PLoS One 7:e34493.

47. Taieb G, Renard D, Thouvenot E, Servillo G, Castelnovo G (2014) Transient punctuate enhancing lesions preceding natalizumab-associated progressive multifocal leukoencephalopathy. J Neurol Sci 346:364-365.

48. Thaker AA, Schmitt SE, Pollard JR, Dubroff JG (2014) Natalizumab-induced progressive multifocal leukoencephalopathy. Clin Nucl Med 39:e365-e366.

49. Tortorella C, Direnzo V, D'Onghia M, Trojano M (2013) Brainstem PML lesion mimicking MS plaque in a natalizumab-treated MS patient. Neurology 81:1470-1471.

50. Travasarou M, Marousi S, Papageorgiou E, Karageorgiou CE (2013) JCV-negative natalizumab-associated progressive multifocal leukoencephalopathy: a clinico-radiological diagnosis. Clin Neurol Neurosurg 115:827-829.

51. Van AG, Van RM, Sciot R, Dubois B, Vermeire S, Noman M, Verbeeck J, Geboes K, Robberecht W, Rutgeerts P (2005) Progressive multifocal leukoencephalopathy after natalizumab therapy for Crohn's disease. N Engl J Med 353:362-368.

52. van P, V, Bartholome E, Bissay V, Bouquiaux O, Bureau M, Caekebeke J, Debruyne J, Declercq I, Decoo D, Denayer P, De SE, D'hooghe M, Dubois B, Dupuis M, Sankari SE, Geens K, Guillaume D, van LW, Lysandropoulos A, de Noordhout AM, Medaer R, Melin A, Peeters K, Ba RP, Retif C, Seeldrayers P, Symons A, Urbain E, Vanderdonckt P, Van IE, Vanopdenbosch L, Vanroose E, Van WB, Willekens B, Willems C, Sindic C (2014) Safety and efficacy of natalizumab in Belgian multiple sclerosis patients: subgroup analysis of the natalizumab observational program. Acta Neurol Belg 114:167-178.

53. Vennegoor A, Wattjes MP, van Munster ET, Kriekaart RL, van Oosten BW, Barkhof F, Killestein J, Polman CH (2011) Indolent course of progressive multifocal leukoencephalopathy during natalizumab treatment in MS. Neurology 76:574-576.

54. Wattjes MP, Verhoeff L, Zentjens W, Killestein J, van Munster ET, Barkhof F, van Eijk JJ (2013) Punctate lesion pattern suggestive of perivascular inflammation in acute natalizumab-associated progressive multifocal leukoencephalopathy: productive JC virus infection or preclinical PML-IRIS manifestation? J Neurol Neurosurg Psychiatry 84:1176-1177.

55. Wattjes MP, Vennegoor A, Mostert J, van Oosten BW, Barkhof F, Killestein J (2014) Diagnosis of asymptomatic natalizumab-associated PML: are we between a rock and a hard place? J Neurol 261:1139-1143.

56. Wenning W, Haghikia A, Laubenberger J, Clifford DB, Behrens PF, Chan A, Gold R (2009) Treatment of progressive multifocal leukoencephalopathy associated with natalizumab. N Engl J Med 361:1075-1080.

57. Wuthrich C, Popescu BF, Gheuens S, Marvi M, Ziman R, Denq SP, Tham M, Norton E, Parisi JE, Dang X, Lucchinetti CF, Koralnik IJ (2013) Natalizumab-associated progressive multifocal leukoencephalopathy in a patient with multiple sclerosis: a postmortem study. J Neuropathol Exp Neurol 72:1043-1051.

58. Zivadinov R, Dwyer MG, Hussein S, Carl E, Kennedy C, Andrews M, Hojnacki D, Heininen-Brown M, Willis L, Cherneva M, Bergsland N, Weinstock-Guttman B (2012) Voxel-wise magnetization transfer imaging study of effects of natalizumab and IFNbeta-1a in multiple sclerosis. Mult Scler 18:1125-1134.

59. Al-Tawfiq JA, Banda RW, Daabil RA, Dawamneh MF (2015) Progressive multifocal leukoencephalopathy (PML) in a patient with lymphoma treated with rituximab: A case report and literature review. J Infect Public Health 8:493-497.

60. Belhassen-Garcia M, Rabano-Gutierrez A, Velasco-Tirado V, Romero-Alegria A, Perez-Garcia ML, Martin-Oterino JA (2015) Atypical progressive multifocal leukoencephalopathy in a patient with antisynthetase syndrome. Intern Med 54:519-524.

61. Bruggemann N, Gottschalk S, Kortke D, Marxsen JH, Moser A (2012) Excessively increased CSF tau in progressive multifocal leukoencephalopathy. Clin Neurol Neurosurg 114:762-764.

62. Coppo P, Laporte JP, Aoudjhane M, Lebon P, Isnard F, Lesage S, Gorin NC, Najman A (1999) Progressive multifocal leucoencephalopathy with peripheral demyelinating neuropathy after autologous bone marrow transplantation for acute myeloblastic leukemia (FAB5). Bone Marrow Transplant 23:401-403.

63. Daibata M, Hatakeyama N, Kamioka M, Nemoto Y, Hiroi M, Miyoshi I, Taguchi H (2001) Detection of human herpesvirus 6 and JC virus in progressive multifocal leukoencephalopathy complicating follicular lymphoma. Am J Hematol 67:200-205.

64. Dima D, Tomuleasa C, Irimie A, Florian IS, Petrushev B, Berindan-Neagoe I, Cucuianu A (2014) Magnetic resonance imaging-based diagnosis of progressive multifocal leukoencephalopathy in a patient with non-Hodgkin lymphoma after therapy with cyclophosphamide, doxorubicin, vincristine, prednisone, and rituximab. Cancer 120:4005-4006.

65. Felli V, Di SA, Anselmi M, Gennarelli A, Sucapane P, Splendiani A, Catalucci A, Marini C, Gallucci M (2014) Progressive Multifocal Leukoencephalopathy Following Treatment with Rituximab in an HIV-Negative Patient with Non-Hodgkin Lymphoma. A Case Report and Literature Review. Neuroradiol J 27:657-664.

66. Fleischmann RM (2009) Progressive multifocal leukoencephalopathy following rituximab treatment in a patient with rheumatoid arthritis. Arthritis Rheum 60:3225-3228.

67. Focosi D, Fazzi R, Montanaro D, Emdin M, Petrini M (2007) Progressive multifocal leukoencephalopathy in a haploidentical stem cell transplant recipient: a clinical, neuroradiological and virological response after treatment with risperidone. Antiviral Res 74:156-158.

68. Freim Wahl SG, Folvik MR, Torp SH (2007) Progressive multifocal leukoencephalopathy in a lymphoma patient with complete remission after treatment with cytostatics and rituximab: case report and review of the literature. Clin Neuropathol 26:68-73.

69. Gaman A, Bold A, Gaman G (2011) The unexpected evolution of a case of diffuse large B-cell non-Hodgkin lymphoma. Rom J Morphol Embryol 52:719-722.

70. Goldberg SL, Pecora AL, Alter RS, Kroll MS, Rowley SD, Waintraub SE, Imrit K, Preti RA (2002) Unusual viral infections (progressive multifocal leukoencephalopathy and cytomegalovirus disease) after high-dose chemotherapy with autologous blood stem cell rescue and peritransplantation rituximab. Blood 99:1486-1488.

71. Grinyo J, Charpentier B, Pestana JM, Vanrenterghem Y, Vincenti F, Reyes-Acevedo R, Apanovitch AM, Gujrathi S, Agarwal M, Thomas D, Larsen CP (2010) An integrated safety profile analysis of belatacept in kidney transplant recipients. Transplantation 90:1521-1527.

72. Hopfinger G, Plessl A, Grisold W, Klimpfinger M, Hoftberger R, Bernt R, Mostl M, Waldner R, Pittermann-Hocker E (2008) Progressive multifocal leukoencephalopathy after rituximab in a patient with relapsed follicular lymphoma and low IgG levels and a low CD4+ lymphocyte count. Leuk Lymphoma 49:2367-2369.

73. Kalisch A, Wilhelm M, Erbguth F, Birkmann J (2014) Progressive multifocal leukoencephalopathy in patients with a hematological malignancy: review of therapeutic options. Chemotherapy 60:47-53.

74. Kaufman GP, Aksamit AJ, Klein CJ, Yi ES, Delone DR, Litzow MR (2014) Progressive multifocal leukoencephalopathy: a rare infectious complication following allogeneic hematopoietic cell transplantation (HCT). Eur J Haematol 92:83-87.

75. Kharfan-Dabaja MA, Ayala E, Greene J, Rojiani A, Murtagh FR, Anasetti C (2007) Two cases of progressive multifocal leukoencephalopathy after allogeneic hematopoietic cell transplantation and a review of the literature. Bone Marrow Transplant 39:101-107.

76. Khoury S, Shapira S, Zilberman T, Mekori YA, Hershko AY (2013) Progressive multifocal leukoencephalopathy in an HIV-negative patient following treatment with rituximab. Isr Med Assoc J 15:321-322.

77. Kranick SM, Mowry EM, Rosenfeld MR (2007) Progressive multifocal leukoencephalopathy after rituximab in a case of non-Hodgkin lymphoma. Neurology 69:704-706.

78. Lejniece S, Murovska M, Chapenko S, Breiksa B, Jaunmuktane Z, Feldmane L, Ziedina I, Gomez-Roman J, Garcia-Cabeza M, Lejnieks A (2011) Progressive multifocal leukoencephalopathy following fludarabine treatment in a chronic lymphocytic leukemia patient. Exp Oncol 33:239-241.

79. Lobo LJ, Reynolds JM, Snyder LD (2013) Rituximab-associated progressive multifocal leukoencephalopathy after lung transplantation. J Heart Lung Transplant 32:752-753.

80. Loyaga-Rendon RY, Taylor DO, Koval CE (2013) Progressive multifocal leukoencephalopathy in a heart transplant recipient following rituximab therapy for antibody-mediated rejection. Am J Transplant 13:1075-1079.

81. Matteucci P, Magni M, Di NM, Carlo-Stella C, Uberti C, Gianni AM (2002) Leukoencephalopathy and papovavirus infection after treatment with chemotherapy and anti-CD20 monoclonal antibody. Blood 100:1104-1105.

82. Moreno C, Montillo M, Panayiotidis P, Dimou M, Bloor A, Dupuis J, Schuh A, Norin S, Geisler C, Hillmen P, Doubek M, Trneny M, Obrtlikova P, Laurenti L, Stilgenbauer S, Smolej L, Ghia P, Cymbalista F, Jaeger U, Stamatopoulos K, Stavroyianni N, Carrington P, Zouabi H, Leblond V, Gomez-Garcia JC, Rubio M, Marasca R, Musuraca G, Rigacci L, Farina L, Paolini R, Pospisilova S, Kimby E, Bradley C, Montserrat E (2015) Ofatumumab in poor-prognosis chronic lymphocytic leukemia: a phase IV, non-interventional, observational study from the European Research Initiative on Chronic Lymphocytic Leukemia. Haematologica 100:511-516.

83. O'Shaughnessy D, Goldman JM, Roddie M, Schofield JB (1994) Dizziness and confusion after bone marrow transplantation. BMJ 309:262-265.

84. Paues J, Vrethem M (2010) Fatal progressive multifocal leukoencephalopathy in a patient with non-Hodgkin lymphoma treated with rituximab. J Clin Virol 48:291-293.

85. Pelosini M, Focosi D, Rita F, Galimberti S, Caracciolo F, Benedetti E, Papineschi F, Petrini M (2008) Progressive multifocal leukoencephalopathy: report of three cases in HIV-negative hematological patients and review of literature. Ann Hematol 87:405-412.

86. Re D, Bamborschke S, Feiden W, Schroder R, Lehrke R, Diehl V, Tesch H (1999) Progressive multifocal leukoencephalopathy after autologous bone marrow transplantation and alpha-interferon immunotherapy. Bone Marrow Transplant 23:295-298.

87. Reddy N, Abel TW, Jagasia M, Morgan D, Weaver K, Greer J (2009) Progressive multifocal leukoencephalopathy in a patient with follicular lymphoma treated with multiple courses of rituximab. Leuk Lymphoma 50:460-462.

88. Rey J, Belmecheri N, Bouayed N, Ivanov V, Coso D, Gastaut JA, Bouabdallah R (2007) JC papovavirus leukoencephalopathy after first line treatment with CHOP and rituximab. Haematologica 92:e101.

89. Ripellino P, Comi C, Mula M, Varrasi C, Conconi A, Stecco A, Brustia D, Nasuelli N, Savio K, De PL, Cantello R, Gaidano G, Monaco F (2011) Progressive multifocal leucoencephalopathy after autologous bone marrow transplantation: a treatment option. BMJ Case Rep 2011.

90. Sanders JS, Riezebos-Brilman A, Homan van der Heide JJ (2012) Progressive multifocal leuko-encephalopathy after ABO-incompatible kidney transplantation. Transpl Int 25:e104-e105.

91. Sano Y, Nakano Y, Omoto M, Takao M, Ikeda E, Oga A, Nakamichi K, Saijo M, Maoka T, Sano H, Kawai M, Kanda T (2015) Rituximab-associated progressive multifocal leukoencephalopathy derived from non-Hodgkin lymphoma: neuropathological findings and results of mefloquine treatment. Intern Med 54:965-970.

92. Sikkema T, Schuiling WJ, Hoogendoorn M (2013) Progressive multifocal leukoencephalopathy during treatment with rituximab and CHOP chemotherapy in a patient with a diffuse large B-cell lymphoma. BMJ Case Rep 2013.

93. Tuccori M, Focosi D, Maggi F, Cosottini M, Meini B, Lena F, Blandizzi C, Del TM, Petrini M (2010) Progressive multifocal leukoencephalopathy: a report of three cases in HIV-negative patients with non-Hodgkin's lymphomas treated with rituximab. Ann Hematol 89:519-522.

94. Vidarsson B, Mosher DF, Salamat MS, Isaksson HJ, Onundarson PT (2002) Progressive multifocal leukoencephalopathy after fludarabine therapy for low-grade lymphoproliferative disease. Am J Hematol 70:51-54.

95. Yokoyama H, Watanabe T, Maruyama D, Kim SW, Kobayashi Y, Tobinai K (2008) Progressive multifocal leukoencephalopathy in a patient with B-cell lymphoma during rituximab-containing chemotherapy: case report and review of the literature. Int J Hematol 88:443-447.

96. Dawson DM (1982) Progressive multifocal leukoencephalopathy in myasthenia gravis. Ann Neurol 11:218-219.

97. Egan JD, Ring BL, Reding MJ, Wells IC, Shuman RM (1980) Reticulum cell sarcoma and progressive multifocal leukoencephalopathy following renal transplantation. Transplantation 29:84-86.

98. Garcia JH, Pearson J, Bonnin J, Gupta KL (1985) Deteriorating neurologic function in a 28-year-old renal transplant recipient. Ala J Med Sci 22:208-214.

99. Gedizlioglu M, Coban P, Ce P, Sivasli IE (2009) An unusual complication of immunosuppression in myasthenia gravis: progressive multifocal leukoencephalopathy. Neuromuscul Disord 19:155-157.

100. Govindappa V, Hicks S, Wichter M, Jolly M (2007) Progressive multifocal leukoencephalopathy in systemic lupus erythematosus. Arthritis Rheum 57:352-354.

101. Ho K, Garancis JC, Paegle RD, Gerber MA, Borkowski WJ (1980) Progressive multifocal leukoencephalopathy and malignant lymphoma of the brain in a patient with immunosuppressive therapy. Acta Neuropathol 52:81-83.

102. Itoh K, Kano T, Nagashio C, Mimori A, Kinoshita M, Sumiya M (2006) Progressive multifocal leukoencephalopathy in patients with systemic lupus erythematosus. Arthritis Rheum 54:1020-1022.

103. Koralnik IJ, Schellingerhout D, Frosch MP (2004) Case records of the Massachusetts General Hospital. Weekly clinicopathological exercises. Case 14-2004. A 66-year-old man with progressive neurologic deficits. N Engl J Med 350:1882-1893.

104. Manz HJ, Dinsdale HB, Morrin PA (1971) Progressive multifocal leukoencephalopathy after renal transplantation. Demonstration of Papova-like virions. Ann Intern Med 75:77-81.

105. McCormick WF, Schochet SS, Jr., Sarles HE, Calverley JR (1976) Progressive multifocal leukoencephalopathy in renal transplant recipients. Arch Intern Med 136:829-834.

106. Nived O, Bengtsson AA, Jonsen A, Sturfelt G (2008) Progressive multifocal leukoencephalopathy - the importance of early diagnosis illustrated in four cases. Lupus 17:1036-1041.

107. Ohta K, Obara K, Sakauchi M, Obara K, Takane H, Yogo Y (2001) Lesion extension detected by diffusion-weighted magnetic resonance imaging in progressive multifocal leukoencephalopathy. J Neurol 248:809-811.

108. Pagnoux C, Hayem G, Roux F, Rouidi SA, Palazzo E, Henin D, Meyer O (2003) JC virus leukoencephalopathy complicating Wegener's granulomatosis. Joint Bone Spine 70:376-379.

109. Reznik M, Halleux J, Urbain E, Mouchette R, Castermans P, Beaujean M (1981) Two cases of progressive multifocal leukoencephalopathy after renal transplantation. Acta Neuropathol Suppl 7:189-191.

110. White RP, Abraham S, Singhal S, Manji H, Clarke CR (2002) Progressive multifocal leucoencephalopathy isolated to the posterior fossa in a patient with systemic lupus erythematosus. Rheumatology (Oxford) 41:826-827.

111. (1995) Case records of the Massachusetts General Hospital. Weekly clinicopathological exercises. Case 20-1995. A 66-year-old man with a history of rheumatoid arthritis treated with adrenocorticosteroids, with the development of aphasia and right-sided weakness. N Engl J Med 332:1773-1780.

112. Beppu M, Kawamoto M, Nukuzuma S, Kohara N (2012) Mefloquine improved progressive multifocal leukoencephalopathy in a patient with systemic lupus erythematosus. Intern Med 51:1245-1247.

113. Jones HR, Jr., Hedley-Whyte ET, Freidberg SR, Kelleher JE, Jr., Krolikowski J (1982) Primary cerebellopontine progressive multifocal leukoencephalopathy diagnosed premortem by cerebellar biopsy. Ann Neurol 11:199-202.

114. Kinoshita M, Iwana K, Shinoura H, Aotsuka S, Sumiya M (1998) Progressive multifocal leukoencephalopathy resembling central nervous system systemic lupus erythematosus. Clin Exp Rheumatol 16:313-315.

115. Malas D, Weiss S (1977) Progressive multifocal leukoencephalopathy and cryptococcal meningitis with systemic lupus erythematosus and thymoma. Ann Neurol 1:188-191.

116. Marriott PJ, O'Brien MD, Mackenzie IC, Janota I (1975) Progressive multifocal leucoencephalopathy: remission with cytarabine. J Neurol Neurosurg Psychiatry 38:205-209.

117. Newton P, Aldridge RD, Lessells AM, Best PV (1986) Progressive multifocal leukoencephalopathy complicating systemic lupus erythematosus. Arthritis Rheum 29:337-343.

118. Shin JW, Jung KH, Lee ST, Moon J, Lim JA, Byun JI, Park KI, Lee SK, Chu K (2014) Mefloquine improved progressive multifocal leukoencephalopathy in a patient with immunoglobulin A nephropathy. J Clin Neurosci 21:1661-1664.

119. Tomura N, Watanabe M, Kato T, Nishino K, Kowada M (1994) Case report: progressive multifocal leukoencephalopathy with prominent medullary veins on angiogram. Clin Radiol 49:66-68.

120. Tubridy N, Wells C, Lewis D, Schon F (2000) Unsuccessful treatment with cidofovir and cytarabine in progressive multifocal leukoencephalopathy associated with dermatomyositis. J R Soc Med 93:374-375.

121. Volker HU, Kraft K, Arnold E, Steinhoff S, Kolios G, Sommer S (2007) Progressive multifocal leukoencephalopathy developing in advanced pulmonal sarcoidosis. Clin Neurol Neurosurg 109:624-630.

122. Vulliemoz S, Lurati-Ruiz F, Borruat FX, Delavelle J, Koralnik IJ, Kuntzer T, Bogousslavsky J, Picard F, Landis T, Du Pasquier RA (2006) Favourable outcome of progressive multifocal leucoencephalopathy in two patients with dermatomyositis. J Neurol Neurosurg Psychiatry 77:1079-1082.

123. Cei M, Mumoli N, Ferrito G, Scazzeri F (2010) Progressive multifocal leukoencephalopathy. South Med J 103:1074-1075.

124. Choy DS, Weiss A, Lin PT (1992) Progressive multifocal leukoencephalopathy following treatment for Wegener's granulomatosis. JAMA 268:600-601.

125. Cuevas LA, Fuchs HA (2004) Progressive multifocal leucoencephalopathy and immunosuppression. Ann Rheum Dis 63:112-113.

126. Epker JL, van BP, van Daele PL, van GT, Vossen A, van Saase JL (2009) Progressive multifocal leukoencephalopathy, a review and an extended report of five patients with different immune compromised states. Eur J Intern Med 20:261-267.

127. Fianchi L, Colosimo C, De LA, Pompucci A, Cattani P, Voso MT, LaRocca LM, Leone G, Pagano L (2010) Atypical presentation of progressive multifocal leukoencephalopathy in a multiple myeloma patient after auto-SCT successfully treated with combination therapy. Bone Marrow Transplant 45:1668-1670.

128. Morgenstern LB, Pardo CA (1995) Progressive multifocal leukoencephalopathy complicating treatment for Wegener's granulomatosis. J Rheumatol 22:1593-1595.

129. Osorio S, de la Camara R, Golbano N, Marti E, Fedele CG, Nieto S, Manzanares R, Fernandez-Ranada JM (2002) Progressive multifocal leukoencephalopathy after stem cell transplantation, unsuccessfully treated with cidofovir. Bone Marrow Transplant 30:963-966.

130. Pugnet G, Pagnoux C, Bezanahary H, Ly KH, Vidal E, Guillevin L (2013) Progressive multifocal encephalopathy after cyclophosphamide in granulomatosis with polyangiitis (Wegener) patients: case report and review of literature. Clin Exp Rheumatol 31:S62-S64.

131. Saxton CR, Gailiunas P, Jr., Helderman JH, Farkas RA, McCoy R, Diehl J, Sagalowsky A, Murphy FK, Ross ED, Silva FR, . (1984) Progressive multifocal leukoencephalopathy in a renal transplant recipient. Increased diagnostic sensitivity of computed tomographic scanning by double-dose contrast with delayed films. Am J Med 77:333-337.

132. Bartsch T, Rempe T, Wrede A, Leypoldt F, Bruck W, Adams O, Rohr A, Jansen O, Wuthrich C, Deuschl G, Koralnik IJ (2015) Progressive neurologic dysfunction in a psoriasis patient treated with dimethyl fumarate. Ann Neurol 78:501-514.

133. Buttmann M, Stoll G (2013) Case reports of PML in patients treated for psoriasis. N Engl J Med 369:1081.

134. Dammeier N, Schubert V, Hauser TK, Bornemann A, Bischof F (2015) Case report of a patient with progressive multifocal leukoencephalopathy under treatment with dimethyl fumarate. BMC Neurol 15:108.

135. Ermis U, Weis J, Schulz JB (2013) PML in a patient treated with fumaric acid. N Engl J Med 368:1657-1658.

136. Hoepner R, Faissner S, Klasing A, Schneider R, Metz I, Bellenberg B, Lukas C, Altmeyer P, Gold R, Chan A (2015) Progressive multifocal leukoencephalopathy during fumarate monotherapy of psoriasis. Neurol Neuroimmunol Neuroinflamm 2:e85.

137. Nieuwkamp DJ, Murk JL, van Oosten BW, Cremers CH, Killestein J, Viveen MC, Van HW, Frijlink DW, Wattjes MP (2015) PML in a patient without severe lymphocytopenia receiving dimethyl fumarate. N Engl J Med 372:1474-1476.

138. Rosenkranz T, Novas M, Terborg C (2015) PML in a patient with lymphocytopenia treated with dimethyl fumarate. N Engl J Med 372:1476-1478.

139. Stoppe M, Thoma E, Liebert UG, Major EO, Hoffmann KT, Classen J, Then BF (2014) Cerebellar manifestation of PML under fumarate and after efalizumab treatment of psoriasis. J Neurol 261:1021-1024.

140. van Oosten BW, Killestein J, Barkhof F, Polman CH, Wattjes MP (2013) PML in a patient treated with dimethyl fumarate from a compounding pharmacy. N Engl J Med 368:1658-1659.

141. Cid J, Revilla M, Cervera A, Cervantes F, Munoz E, Ferrer I, Montserrat E (2000) Progressive multifocal leukoencephalopathy following oral fludarabine treatment of chronic lymphocytic leukemia. Ann Hematol 79:392-395.

142. Gonzalez H, Bolgert F, Camporo P, Leblond V (1999) Progressive multifocal leukoencephalitis (PML) in three patients treated with standard-dose fludarabine (FAMP). Hematol Cell Ther 41:183-186.

143. Saad ED, Thomas DA, O'Brien S, Fuller GN, Medeiros LJ, Forman A, Albitar M, Schomer D, Kantarjian HM, Keating MJ (2000) Progressive multifocal leukoencephalopathy with concurrent Richter's syndrome. Leuk Lymphoma 38:183-190.

144. Saumoy M, Castells G, Escoda L, Mares R, Richart C, Ugarriza A (2002) Progressive multifocal leukoencephalopathy in chronic lymphocytic leukemia after treatment with fludarabine. Leuk Lymphoma 43:433-436.

145. Zabernigg A, Maier H, Thaler J, Gattringer C (1994) Late-onset fatal neurological toxicity of fludarabine. Lancet 344:1780.

146. Clifford DB, Ances B, Costello C, Rosen-Schmidt S, Andersson M, Parks D, Perry A, Yerra R, Schmidt R, Alvarez E, Tyler KL (2011) Rituximab-associated progressive multifocal leukoencephalopathy in rheumatoid arthritis. Arch Neurol 68:1156-1164.

147. Harris HE (2008) Progressive multifocal leucoencephalopathy in a patient with systemic lupus erythematosus treated with rituximab. Rheumatology (Oxford) 47:224-225.

148. Kobayashi Z, Akaza M, Numasawa Y, Ishihara S, Tomimitsu H, Nakamichi K, Saijo M, Morio T, Shimizu N, Sanjo N, Shintani S, Mizusawa H (2013) Failure of mefloquine therapy in progressive multifocal leukoencephalopathy: report of two Japanese patients without human immunodeficiency virus infection. J Neurol Sci 324:190-194.

149. Ng C, Slavin MA, Seymour JF (2003) Progressive multifocal leukoencephalopathy complicating Waldenstrom's macroglobulinaemia. Leuk Lymphoma 44:1819-1821.

150. Carson KR, Newsome SD, Kim EJ, Wagner-Johnston ND, von GG, Moskowitz CH, Moskowitz AJ, Rook AH, Jalan P, Loren AW, Landsburg D, Coyne T, Tsai D, Raisch DW, Norris LB, Bookstaver PB, Sartor O, Bennett CL (2014) Progressive multifocal leukoencephalopathy associated with brentuximab vedotin therapy: a report of 5 cases from the Southern Network on Adverse Reactions (SONAR) project. Cancer 120:2464-2471.

151. von GG, Pardo CA, Calabresi PA, Newsome SD (2012) PML-IRIS in a patient treated with brentuximab. Neurology 79:2075-2077.

152. Wagner-Johnston ND, Bartlett NL, Cashen A, Berger JR (2012) Progressive multifocal leukoencephalopathy in a patient with Hodgkin lymphoma treated with brentuximab vedotin. Leuk Lymphoma 53:2283-2286.

153. D'Souza A, Wilson J, Mukherjee S, Jaiyesimi I (2010) Progressive multifocal leukoencephalopathy in chronic lymphocytic leukemia: a report of three cases and review of the literature. Clin Lymphoma Myeloma Leuk 10:E1-E9.

154. del Pilar MM, Cravens PD, Winger R, Kieseier BC, Cepok S, Eagar TN, Zamvil SS, Weber MS, Frohman EM, Kleinschmidt-DeMasters BK, Montine TJ, Hemmer B, Marra CM, Stuve O (2009) Depletion of B lymphocytes from cerebral perivascular spaces by rituximab. Arch Neurol 66:1016-1020.

155. Desmond R, Lynch K, Gleeson M, Farrell M, Murphy P (2010) Progressive multifocal leukencephalopathy and cerebral toxoplasmosis in a patient with CLL. Am J Hematol 85:607.

156. Garrote H, de la Fuente A, Ona R, Rodriguez I, Echevarria JE, Sepulveda JM, Garcia JF (2015) Long-term survival in a patient with progressive multifocal leukoencephalopathy after therapy with rituximab, fludarabine and cyclophosphamide for chronic lymphocytic leukemia. Exp Hematol Oncol 4:8.

157. Herold T, Seiler T, Egensperger R, Trumm C, Bergmann M, Franke D, Mumm FF, Schinwald N, Buske C, Dreyling M (2012) Progressive multifocal leukoencephalopathy after treatment with rituximab, fludarabine and cyclophosphamide in a patient with chronic lymphocytic leukemia. Leuk Lymphoma 53:169-172.

158. Smolle E, Trojan A, Schuster SJ, Haybaeck J (2014) Progressive multifocal leukoencephalopathy--a case report and review of the literature. In Vivo 28:941-948.

159. Belli LS, De CL, Romani F, Rondinara GF, Rimoldi P, Alberti A, Bettale G, Dughetti L, Ideo G, Sberna M, . (1993) Dysarthria and cerebellar ataxia: late occurrence of severe neurotoxicity in a liver transplant recipient. Transpl Int 6:176-178.

160. Bronster DJ, Lidov MW, Wolfe D, Schwartz ME, Miller CM (1995) Progressive multifocal leukoencephalopathy after orthotopic liver transplantation. Liver Transpl Surg 1:371-372.

161. Flomenbaum MA, Jarcho JA, Schoen FJ (1991) Progressive multifocal leukoencephalopathy fifty-seven months after heart transplantation. J Heart Lung Transplant 10:888-893.

162. Hall WA, Martinez AJ, Dummer JS (1988) Progressive multifocal leukoencephalopathy after cardiac transplantation. Neurology 38:995-996.

163. Ouwens JP, Haaxma-Reiche H, Verschuuren EA, Timens W, Steenhuis LH, de Boer WJ, van der Bij W (2000) Visual symptoms after lung transplantation: a case of progressive multifocal leukoencephalopathy. Transpl Infect Dis 2:29-32.

164. Lefevre G, Queyrel V, Maurage CA, Laurent C, Launay D, Lacour A, Charlanne H, Morell-Dubois S, Lambert M, Maillard H, Vermersch P, Hachulla E, Hatron PY (2009) Effective immune restoration after immunosuppressant discontinuation in a lupus patient presenting progressive multifocal leukoencephalopathy. J Neurol Sci 287:246-249.

165. Manfro RC, Vedolin L, Cantarelli M, Oppitz P, Antunes AC, Rieder CR (2009) Progressive multifocal leukoencephalopathy in a kidney transplant recipient after conversion to mycophenolic acid therapy. Transpl Infect Dis 11:189-190.

166. Nagayama S, Gondo Y, Araya S, Minato N, Fujita-Nakata M, Kaito M, Nakanishi M, Tanaka K, Yamaya H, Yokoyama H, Nakamichi K, Saijo M, Okamoto K, Toyoshima Y, Kakita A, Matsui M (2013) Progressive multifocal leukoencephalopathy developed 26 years after renal transplantation. Clin Neurol Neurosurg 115:1482-1484.

167. Pavlovic AM, Bonaci-Nikolic B, Kozic D, Ostojic J, Abinun M, Svabic-Medjedovic T, Nikolic M, Sternic N (2012) Progressive multifocal leukoencephalopathy associated with mycophenolate mofetil treatment in a woman with lupus and CD4+ T-lymphocyte deficiency. Lupus 21:100-102.

168. Ahmed F, Aziz T, Kaufman LD (1999) Progressive multifocal leukoencephalopathy in a patient with systemic lupus erythematosus. J Rheumatol 26:1609-1612.

169. Mesquita R, Parravicini C, Bjorkholm M, Ekman M, Biberfeld P (1992) Macrophage association of polyomavirus in progressive multifocal leukoencephalopathy: an immunohistochemical and ultrastructural study. Case report. APMIS 100:993-1000.

170. Vollmer-Haase J, Young P, Ringelstein EB (1997) Efficacy of camptothecin in progressive multifocal leucoencephalopathy. Lancet 349:1366.

171. Aksamit AJ, Jr., de Groen PC (1995) Cyclosporine-related leukoencephalopathy and PML in a liver transplant recipient. Transplantation 60:874-876.

172. Berner B, Krieter DH, Rumpf KW, Grunewald RW, Beuche W, Weber T, Muller GA (1999) Progressive multifocal leukoencephalopathy in a renal transplant patient diagnosed by JCV-specific DNA amplification and an intrathecal humoral immune response to recombinant virus protein 1. Nephrol Dial Transplant 14:462-465.

173. Gentile S, Sacerdote I, Roccatello D, Giordana MT (1996) Progressive multifocal leukoencephalopathy during cyclosporine treatment. A case report. Ital J Neurol Sci 17:363-366.

174. Lewis AR, Kline LB, Pinkard NB (1993) Visual loss due to progressive multifocal leukoencephalopathy in a heart transplant patient. J Clin Neuroophthalmol 13:237-241.

175. Dastmalchi M, Laki J, Lundberg IE, Iacobaeus E (2012) Progressive multifocal leukoencephalopathy in a patient with polymyositis: case report and literature review. J Rheumatol 39:1299-1303.

176. Owczarczyk K, Hilker R, Brunn A, Hallek M, Rubbert A (2007) Progressive multifocal leucoencephalopathy in a patient with sarcoidosis--successful treatment with cidofovir and mirtazapine. Rheumatology (Oxford) 46:888-890.

177. Rankin E, Scaravilli F (1995) Progressive multifocal leukoencephalopathy in a patient with rheumatoid arthritis and polymyositis. J Rheumatol 22:777-779.

178. Shprecher D, Frech T, Chin S, Eskandari R, Steffens J (2008) Progressive multifocal leucoencephalopathy associated with lupus and methotrexate overdose. Lupus 17:1029-1032.

179. Martin SI, Marty FM, Fiumara K, Treon SP, Gribben JG, Baden LR (2006) Infectious complications associated with alemtuzumab use for lymphoproliferative disorders. Clin Infect Dis 43:16-24.

180. Uppenkamp M, Engert A, Diehl V, Bunjes D, Huhn D, Brittinger G (2002) Monoclonal antibody therapy with CAMPATH-1H in patients with relapsed high- and low-grade non-Hodgkin's lymphomas: a multicenter phase I/II study. Ann Hematol 81:26-32.

181. Arbusow V, Strupp M, Pfister HW, Seelos KC, Bruckmann H, Brandt T (2000) Contrast enhancement in progressive multifocal leukoencephalopathy: a predictive factor for long-term survival? J Neurol 247:306-308.

182. Sponzilli EE, Smith JK, Malamud N, McCulloch JR (1975) Progressive multifocal leukoencephalopathy: a complication of immunosuppressive treatment. Neurology 25:664-668.

183. Matijaca M, Vlasic-Matas J, Jankovic S, Pintaric I, Marovic A (2007) Neurotoxicity that may mimic progressive multifocal leukoencephalopathy in patient with transplanted kidney. Coll Antropol 31:349-353.

184. Phillips T, Jacobs R, Ellis EN (2004) Polyoma nephropathy and progressive multifocal leukoencephalopathy in a renal transplant recipient. J Child Neurol 19:301-304.

185. Kothary N, Diak IL, Brinker A, Bezabeh S, Avigan M, Dal PG (2011) Progressive multifocal leukoencephalopathy associated with efalizumab use in psoriasis patients. J Am Acad Dermatol 65:546-551.

186. Embrey JR, Silva FG, Helderman JH, Peters PC, Sagalowsky AI (1988) Long-term survival and late development of bladder cancer in renal transplant patient with progressive multifocal leukoencephalopathy. J Urol 139:580-581.

187. ZuRhein GM, Varakis J (1974) Letter: Progressive multifocal leukoencephalopathy in a renal-allograft recipient. N Engl J Med 291:798.

188. Fredericks CA, Kvam KA, Bear J, Crabtree GS, Josephson SA (2014) A case of progressive multifocal leukoencephalopathy in a lupus patient treated with belimumab. Lupus 23:711-713.

189. Leblanc-Trudeau C, Masetto A, Bocti C (2015) Progressive multifocal leukoencephalopathy associated with belimumab in a patient with systemic lupus erythematosus. J Rheumatol 42:551-552.

190. Przepiorka D, Jaeckle KA, Birdwell RR, Fuller GN, Kumar AJ, Huh YO, McCutcheon I (1997) Successful treatment of progressive multifocal leukoencephalopathy with low-dose interleukin-2. Bone Marrow Transplant 20:983-987.

191. Seong D, Bruner JM, Lee KH, Mirza N, Kwon BD, Lee JH, Lee YY, Ro J, Talpaz M, Champlin R, Deisseroth AB (1996) Progressive multifocal leukoencephalopathy after autologous bone marrow transplantation in a patient with chronic myelogenous leukemia. Clin Infect Dis 23:402-403.

192. Montoto S, Moreno C, Domingo-Domenech E, Estany C, Oriol A, Altes A, Besalduch J, Pedro C, Gardella S, Escoda L, Asensio A, Vivancos P, Galan P, de Sevilla AF, Ribera JM, Briones J, Colomer D, Campo E, Montserrat E, Lopez-Guillermo A (2008) High clinical and molecular response rates with fludarabine, cyclophosphamide and mitoxantrone in previously untreated patients with advanced stage follicular lymphoma. Haematologica 93:207-214.

193. Rodriguez L, Ribera JM, Batlle M, Xicoy B, Mate JL, Milla F, Feliu E (2002) Progressive multifocal leukoencephalopathy shortly after the diagnosis of follicular lymphoma in a patient treated with fludarabine. Haematologica 87:ECR26.

194. Baehring JM, Vives K, Bannykh S (2007) Progressive multifocal leukoencephalopathy in a patient with marginal zone B-cell lymphoma. J Neurooncol 85:289-290.

195. Rahmlow M, Shuster EA, Dominik J, Deen HG, Jr., Dickson DW, Aksamit AJ, Jr., Robles HA, Freeman WD (2008) Leflunomide-associated progressive multifocal leukoencephalopathy. Arch Neurol 65:1538-1539.

196. Warnatz K, Peter HH, Schumacher M, Wiese L, Prasse A, Petschner F, Vaith P, Volk B, Weiner SM (2003) Infectious CNS disease as a differential diagnosis in systemic rheumatic diseases: three case reports and a review of the literature. Ann Rheum Dis 62:50-57.

197. Lach B, Connolly B, Wuthrich C, Koralnik IJ (2014) Inflammatory infratentorial progressive multifocal leukoencephalopathy in a patient with rheumatoid arthritis. Neuropathology 34:39-44.

198. Marzocchetti A, Wuthrich C, Tan CS, Tompkins T, Bernal-Cano F, Bhargava P, Ropper AH, Koralnik IJ (2008) Rearrangement of the JC virus regulatory region sequence in the bone marrow of a patient with rheumatoid arthritis and progressive multifocal leukoencephalopathy. J Neurovirol 14:455-458.

199. Yehia B, Davison A, Sisson S (2009) Transplant troubles. Am J Med 122:629-631.

200. Crowder CD, Gyure KA, Drachenberg CB, Werner J, Morales RE, Hirsch HH, Ramos E (2005) Successful outcome of progressive multifocal leukoencephalopathy in a renal transplant patient. Am J Transplant 5:1151-1158.

201. Shitrit D, Nirit L, Shiran SI, Izbicki G, Sofer D, Eldad M, Kramer MR (2003) Progressive multifocal leukoencephalopathy in a lung transplant recipient. J Heart Lung Transplant 22:946-950.

202. Boulton-Jones JR, Fraser-Moodie C, Ryder SD (2001) Long term survival from progressive multifocal leucoencephalopathy after liver transplantation. J Hepatol 35:828-829.

203. Lima MA, Hanto DW, Curry MP, Wong MT, Dang X, Koralnik IJ (2005) Atypical radiological presentation of progressive multifocal leukoencephalopathy following liver transplantation. J Neurovirol 11:46-50.

204. Chakraborty S, Tarantolo SR, Treves J, Sambol D, Hauke RJ, Batra SK (2011) Progressive Multifocal Leukoencephalopathy in a HIV-Negative Patient with Small Lymphocytic Leukemia following Treatment with Rituximab. Case Rep Oncol 4:136-142.

205. Marie I, Guegan-Massardier E, Levesque H (2011) Progressive multifocal leukoencephalopathy in refractory polymyositis treated with rituximab. Eur J Intern Med 22:e13-e14.

206. Ray M, Curtis JR, Baddley JW (2014) A case report of progressive multifocal leucoencephalopathy (PML) associated with adalimumab. Ann Rheum Dis 73:1429-1430.

207. Isidoro L, Pires P, Rito L, Cordeiro G (2014) Progressive multifocal leukoencephalopathy in a patient with chronic lymphocytic leukaemia treated with alemtuzumab. BMJ Case Rep 2014.

208. Waggoner J, Martinu T, Palmer SM (2009) Progressive multifocal leukoencephalopathy following heightened immunosuppression after lung transplant. J Heart Lung Transplant 28:395-398.

209. Chihara D, Takeoka T, Shirase T, Kishimoto W, Arimoto-Miyamoto K, Tsuji M, Ohno T (2010) Progressive multifocal leukoencephalopathy in myelodysplastic syndrome involving pure red cell aplasia. Intern Med 49:2347-2352.

210. Klintmalm GB, Feng S, Lake JR, Vargas HE, Wekerle T, Agnes S, Brown KA, Nashan B, Rostaing L, Meadows-Shropshire S, Agarwal M, Harler MB, Garcia-Valdecasas JC (2014) Belatacept-based immunosuppression in de novo liver transplant recipients: 1-year experience from a phase II randomized study. Am J Transplant 14:1817-1827.

211. Warsch S, Hosein PJ, Morris MI, Teomete U, Benveniste R, Chapman JR, Lossos IS (2012) Progressive multifocal leukoencephalopathy following treatment with bendamustine and rituximab. Int J Hematol 96:274-278.

212. Heine A, Schmiedel A, Menschik T, Held SA, Erdmann C, Brossart P (2013) Regression of liver metastases after treatment with oxaliplatin/capecitabine and development of a progressive multifocal leukoencephalopathy in a patient with advanced thymoma. J Clin Oncol 31:e203-e205.

213. Palmieri A, Valentinis L, Bazzano S, Baldi A, Orlando F, Tenaglia S, D'Anna S (2011) Progressive multifocal leukoencephalopathy following chemotherapy for lung cancer. Neurol Sci 32:683-685.

214. Berghoff M, Schanzer A, Hildebrandt GC, Dassinger B, Klappstein G, Kaps M, Gizewski ER, Acker T, Grams A (2013) Development of progressive multifocal leukoencephalopathy in a patient with non-Hodgkin lymphoma 13 years after treatment with cladribine. Leuk Lymphoma 54:1340-1342.

215. Salmaggi A, Maccagnano E, Castagna A, Zeni S, Fantini F, Cinque P, Savoiardo M (2001) Reversal of CSF positivity for JC virus genome by cidofovir in a patient with systemic lupus erythematosus and progressive multifocal leukoencephalopathy. Neurol Sci 22:17-20.

216. Stahl NI (2008) Progressive multifocal leukoencephalopathy in a minimally immunosuppressed patient with systemic lupus erythematosus treated with dapsone. J Rheumatol 35:725-727.

217. Graff-Radford J, Robinson MT, Warsame RM, Matteson EL, Eggers SD, Keegan BM (2012) Progressive multifocal leukoencephalopathy in a patient treated with etanercept. Neurologist 18:85-87.

218. Kiewe P, Seyfert S, Korper S, Rieger K, Thiel E, Knauf W (2003) Progressive multifocal leukoencephalopathy with detection of JC virus in a patient with chronic lymphocytic leukemia parallel to onset of fludarabine therapy. Leuk Lymphoma 44:1815-1818.

219. Kumar D, Bouldin TW, Berger RG (2010) A case of progressive multifocal leukoencephalopathy in a patient treated with infliximab. Arthritis Rheum 62:3191-3195.

220. Weber SC, Uhlenberg B, Raile K, Querfeld U, Muller D (2011) Polyoma virus-associated progressive multifocal leukoencephalopathy after renal transplantation: regression following withdrawal of mycophenolate mofetil. Pediatr Transplant 15:E19-E24.

221. Verhelst X, Vanhooren G, Vanopdenbosch L, Casselman J, Laleman W, Pirenne J, Nevens F, Orlent H (2011) Progressive multifocal leukoencephalopathy in liver transplant recipients: a case report and review of the literature. Transpl Int 24:e30-e34.

222. Steurer M, Clausen J, Gotwald T, Gunsilius E, Stockhammer G, Gastl G, Nachbaur D (2003) Progressive multifocal leukoencephalopathy after allogeneic stem cell transplantation and posttransplantation rituximab. Transplantation 76:435-436.

223. Hasan MM, Taylor P (2005) Progressive multifocal leucoencephalopathy in a case of chronic lymphocytic leukaemia. Br J Haematol 130:808.

224. Worthmann F, Turker T, Muller AR, Patt S, Stoltenburg-Didinger G (1994) Progressive multifocal leukoencephalopathy after orthotopic liver transplantation. Transplantation 57:1268-1271.

225. Kobayashi K, Okamoto Y, Inoue H, Usui T, Ihara M, Kawamata J, Miki Y, Mimori T, Tomimoto H, Takahashi R (2009) Leukoencephalopathy with cognitive impairment following tocilizumab for the treatment of rheumatoid arthritis (RA). Intern Med 48:1307-1309.

226. Owen RG, Patmore RD, Smith GM, Barnard DL (1995) Cytomegalovirus-induced T-cell proliferation and the development of progressive multifocal leucoencephalopathy following bone marrow transplantation. Br J Haematol 89:196-198.

227. Buckanovich RJ, Liu G, Stricker C, Luger SM, Stadtmauer EA, Schuster SJ, Duffy K, Tsai D, Pruitt A, Porter DL (2002) Nonmyeloablative allogeneic stem cell transplantation for refractory Hodgkin's lymphoma complicated by interleukin-2 responsive progressive multifocal leukoencephalopathy. Ann Hematol 81:410-413.

228. McNally PG, Taylor JM, Wood JK (1988) Progressive multifocal leucoencephalopathy associated with chronic lymphocytic leukaemia. Clin Lab Haematol 10:229-233.
